# Identification of novel genes in the carotenogenic and oleaginous yeast *Rhodotorula toruloides* through genome-wide insertional mutagenesis

**DOI:** 10.1186/s12866-018-1151-6

**Published:** 2018-02-21

**Authors:** Yanbin Liu, Chong Mei John Koh, Sihui Amy Yap, Minge Du, Mya Myintzu Hlaing, Lianghui Ji

**Affiliations:** 1 Biomaterials and Biocatalysts Group, Temasek Life Sciences Laboratory, 1 Research Link, National University of Singapore, Singapore, 117604 Singapore; 2 School of Biological Sciences, Nanyang Technological University, 60 Nanyang Drive, Singapore, 637551 Singapore

**Keywords:** *Agrobacterium tumefaciens*-mediated transformation, *Pucciniomycotina*, Insertional mutagenesis, Metabolic engineering, Carotenoid and lipid biosynthesis

## Abstract

**Background:**

*Rhodotorula toruloides* is an outstanding producer of lipids and carotenoids. Currently, information on the key metabolic pathways and their molecular basis of regulation remains scarce, severely limiting efforts to engineer it as an industrial host.

**Results:**

We have adapted *Agrobacterium tumefaciens*-mediated transformation (ATMT) as a gene-tagging tool for the identification of novel genes in *R. toruloides*. Multiple factors affecting transformation efficiency in several species in the *Pucciniomycotina* subphylum were optimized. The *Agrobacterium* transfer DNA (T-DNA) showed predominantly single-copy chromosomal integrations in *R. toruloides*, which were trackable by high efficiency thermal asymmetric interlaced PCR (hiTAIL-PCR). To demonstrate the application of random T-DNA insertions for strain improvement and gene hunting, 3 T-DNA insertional libraries were screened against cerulenin, nile red and tetrazolium violet respectively, resulting in the identification of 22 mutants with obvious phenotypes in fatty acid or lipid metabolism. Similarly, 5 carotenoid biosynthetic mutants were obtained through visual screening of the transformants. To further validate the gene tagging strategy, one of the carotenoid production mutants, RAM5, was analyzed in detail. The mutant had a T-DNA inserted at the putative phytoene desaturase gene *CAR1*. Deletion of *CAR1* by homologous recombination led to a phenotype similar to RAM5 and it could be genetically complemented by re-introduction of the wild-type *CAR1* genome sequence.

**Conclusions:**

T-DNA insertional mutagenesis is an efficient forward genetic tool for gene discovery in *R. toruloides* and related oleaginous yeast species. It is also valuable for metabolic engineering in these hosts. Further analysis of the 27 mutants identified in this study should augment our knowledge of the lipid and carotenoid biosynthesis, which may be exploited for oil and isoprenoid metabolic engineering.

**Electronic supplementary material:**

The online version of this article (10.1186/s12866-018-1151-6) contains supplementary material, which is available to authorized users.

## Background

A large number of oleaginous microorganisms capable of producing more than 20% of their dry biomass as lipids have been reported to date [1–3]. They are potential alternative hosts to plants for the production of lipid and fatty acid derivatives, such as biodiesel, alkane, fatty alcohol and wax [1, 4–7]. On the other hand, only limited number of non-photosynthetic microorganisms can naturally produce carotenoids, which are protective agents against UV radiation and oxidative stress (for review, see [8]). *Rhodotorula toruloides* (syn. *Rhodosporidium toruloides* [9]), a species of the *Pucciniomycotina* subphylum, has gained increasing attention due to its outstanding cell growth rate in high-density fermentation, high lipid and carotenoid productivity, and the capability to utilize cheap feedstocks [10–15].

Genetic tools for *R. toruloides* have increased steadily over recent years since the first report of stable genetic transformation [16]. *R. toruloides* is being developed as a new synthetic biology platform [16–26]. To date, information regarding the molecular control of metabolism and catabolism remains rare in this host, severely limiting the development of *R. toruloides* as an industrial workhorse.

Microbial adaptive laboratory evolution (ALE) is a useful tool for metabolic engineering [27]. Chemical mutagens and ultraviolet radiation are often used to improve strains or populations of interests under a specific selection pressure. Such techniques usually produce mutants with point mutations. Despite the technological advancement of genome sequencing technology, the identification of point mutations remains a tedious task [28, 29]. DNA insertional mutagenesis (IM) has become a versatile forward genetic tool in diverse species, including plants [30], animals [31, 32], algae [33], bacteria [34] and fungi [35]. Due to the high efficiency in generating genetic diversity, IM could be exploited for fast strain improvement, particularly for microbes [36]. Generally, a good gene tagging tool should have the following features: i). DNA is randomly integrated into the nuclear genome [35]; ii). disrupted gene targets can be easily identified [37, 38]; iii). the host contains a haploid genome [39–41]. *Agrobacterium tumefaciens*-mediated transformation (ATMT) delivers the T-DNA into the host’s nuclear genome and has been widely used as an IM tool, particularly in fungi and plants [42–44].

Here, we demonstrate the application of ATMT for gene discovery and modifications of the metabolic pathway in *R. toruloides*.

## Results

### Application of ATMT in *Puicciniomycotina* subphylum

**Fig. 1:**
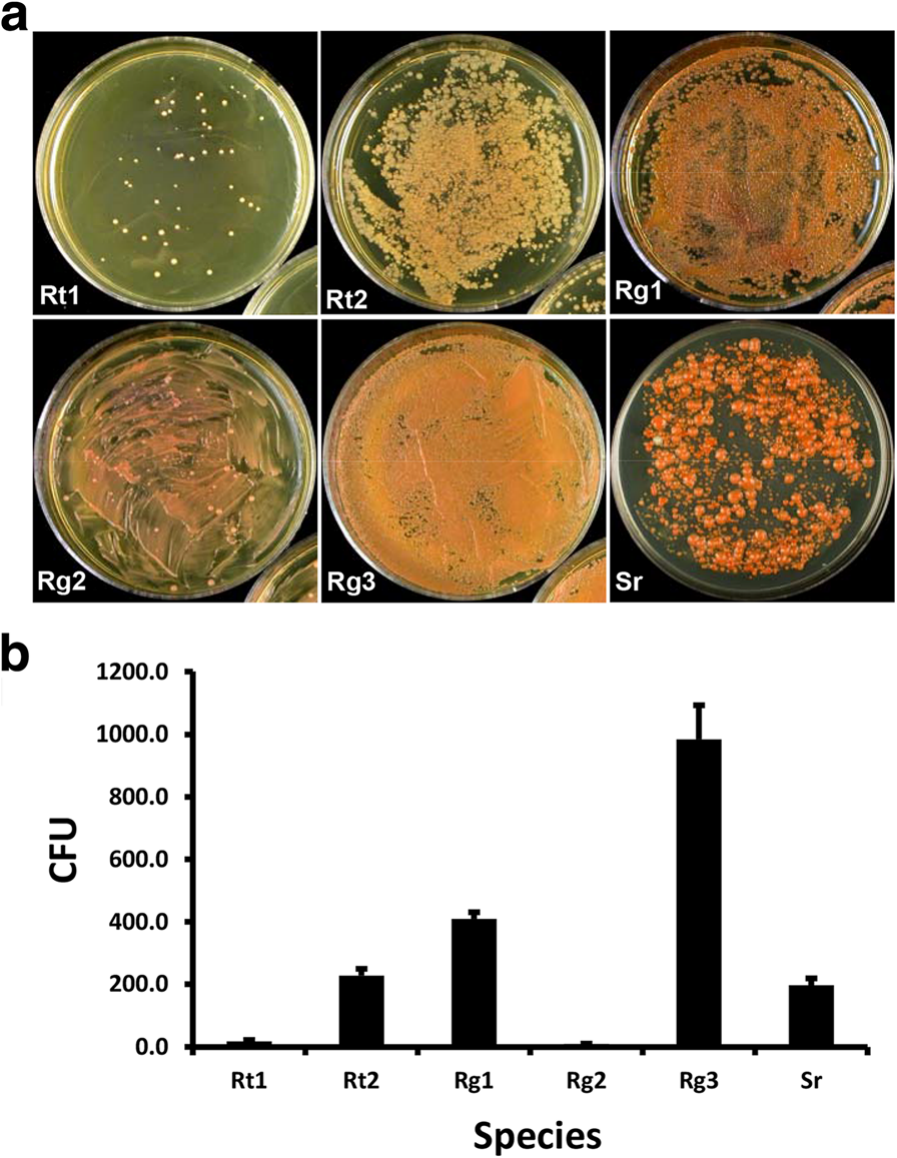
ATMT in *Pucciniomycotina* species. **a** ATMT without the use of membrane support. **b** Transformation efficiency (TFE). *Agrobacterium* culture harboring binary vector pRH201 [16] was used as the donor in all trials. TFE was represented as CFU, colony forming unit per 10^6^ transformed fungal cells. Abbreviations of species: Rt1, *R. toruloides* ATCC 10657; Rt2, *R. toruloides* ATCC 10788; Rg1, *R. glutinis* ATCC 90781; Rg2, *R. glutinis* ATCC 204091; Rg3, *R. graminis* WP1; Sr, *S. roseus*

We have reported a reliable transformation protocol for *R. toruloides* ATCC 10657 (Rt1) using the dominant selection conferred by the codon-optimized hygromycin resistance gene (hygromycin-B-phosphotransferase gene *hpt-3*) [16]. While the method was generally applicable in several species or strains in *Pucciniomycotina*, e.g. *R. toruloides* ATCC 10788 (Rt2), *R. glutinis* ATCC 90781 (Rg1), *R. glutinis* ATCC 204091 (Rg2), *R. graminis* WP1 (Rg3), and *Sporobolomyces roseus* FGSC 10293 (IAM13481, Sr) (Fig. 1a), large variations in the transformation efficiency (TFE, or Colony Forming Unit per 10^6^ fungal cells), were observed (Fig. 1b). The average of CFU for Rg3, Rg1, Rt2 and Sr was 985, 409, 227 and 197, respectively (Fig. 1b). Notably, strains Rt1 and Rg2 showed much lower TFE, producing only 18 and 10 CFU, respectively (Fig. 1b). Colony PCR (data not shown) and Southern blot analysis confirmed that more than 90% transformants contained an integrated T-DNA (Fig. 3; Data on Sr and Rg3 are not shown).

### Optimization of ATMT protocol for large-scale screening

**Fig. 2:**
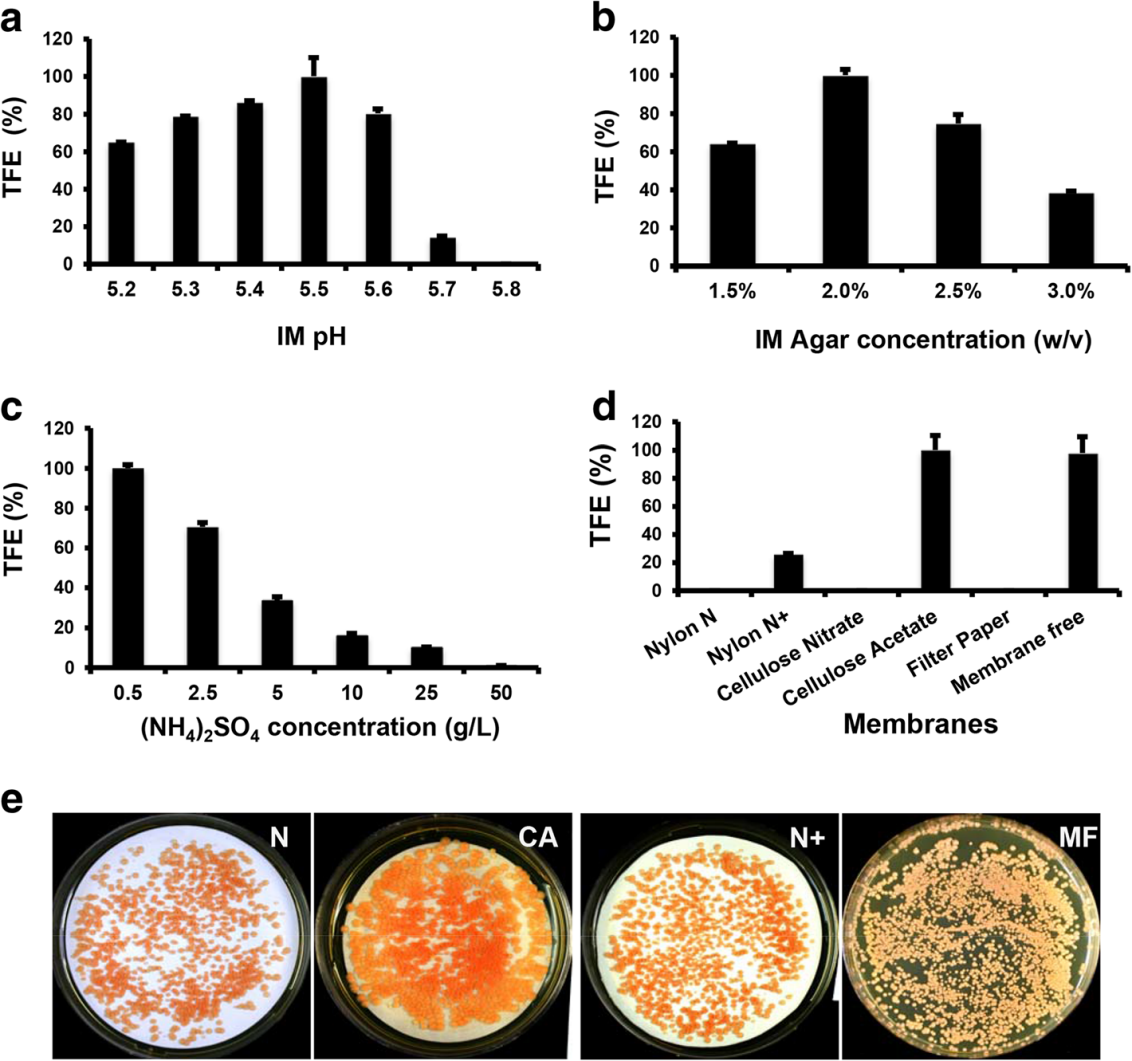
Factors affecting transformation efficiency. **a** pH of induction medium (IM). IM pH was adjusted with 1.0 M hydrogen chloride. **b** Agar concentration of IM. **c** Ammonium sulfate concentration in IM. **d** Different membrane types. **e** Representative transformation on selected membranes. Membrane-free: transformation conducted without supporting membrane. *R. toruloides* ATCC 10657 was used as the T-DNA recipient. TFE is presented as the relative transformation efficiency, where the highest value under each tested condition was set to 100%. Each condition was analyzed in triplicates. Error bars represent standard derivations. The induction medium was pH 5.5. Abbreviations of membranes: N, Nylon Hybond N membrane; CA, Cellulose acetate membrane; N^+^, Nylon Hybond N^+^ membrane; MF, membrane-free

The low TFE for some strains prompted us to investigate the effects of various co-culture parameters. Similar to other reports, virulence inducer (acetosyringone) for agrobacteria, co-culture time, cell ratio between T-DNA recipient and donor, and promoters used to drive *hpt-3* expression drastically influenced the TFE in *R. toruloides* (Additional file 1: Figure S1A-D). Notably, TFE was highly sensitive to the pH of the induction medium (Fig. 2a), where even a slight increase of pH from the optimum (pH 5.5) resulted in a dramatic decrease in TFE (Fig. 2a). The hardness of co-culture medium (agar concentration) also influenced TFE, with the optimal agar concentration observed at 2.0% (*w*/*v*) (Fig. 2b). The role of nitrogen concentration on TFE was investigated due to its multiple effects on energy metabolism, cell growth and differentiation [45, 46]. In our standard ATMT protocol, 0.5 g/L ammonium sulfate was used as the sole nitrogen source in the induction medium [16]. Increasing the concentration of ammonium sulfate led to severe reduction in TFE, and transformation was completely abolished at a level of 50 g/L (Fig. 2c).

Furthermore, the effects of membrane types on TFE were investigated. Results showed that the different supporting membranes dramatically affected TFE, where the positively charged (nylon Hybond N^+^) and neutral membrane (cellulose acetate) supported higher TFE. Interestingly, co-culturing cells directly on the surface of agar medium (without the support of any membrane, membrane-free) led to a high TFE, comparable to that with cellulose acetate membrane (Fig. 2d).

### Characterization of genome-wide T-DNA insertion patterns

**Fig. 3:**
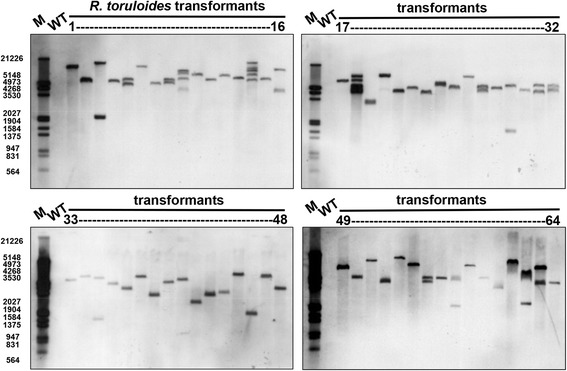
Southern blot analysis of *R. toruloides* transformants. Genomic DNA samples of 64 randomly selected transformants of pRH201 and wild-type strain *R. toruloides* ATCC 10657 (5 μg) were digested with PstI and separated by electrophoresis in 0.8% agarose gel. The 581 bp DNA fragment of partial *hpt-3* gene amplified using oligos HptRU and HptRSL2 was used as the probe (Additional file 5: Table S3). Lane M, DIG-labeled DNA molecular size marker III (Roche Diagnosis, USA). Fragment sizes (bp): 564, 831, 947, 1375, 1584, 1904, 2027, 3530, 4268, 4973, 5148 and 21,226. WT: wild-type strain ATCC 10657

Southern blot analysis of 64 T-DNA mutants from *R. toruloides* ATCC 10657 showed that 75% of transformants contained a single copy of T-DNA, 20% contained two copies, and the rest 5% contained three copies or more (Fig. 3). The average copy number of T-DNA in the genome was 1.36.

**Fig. 4:**
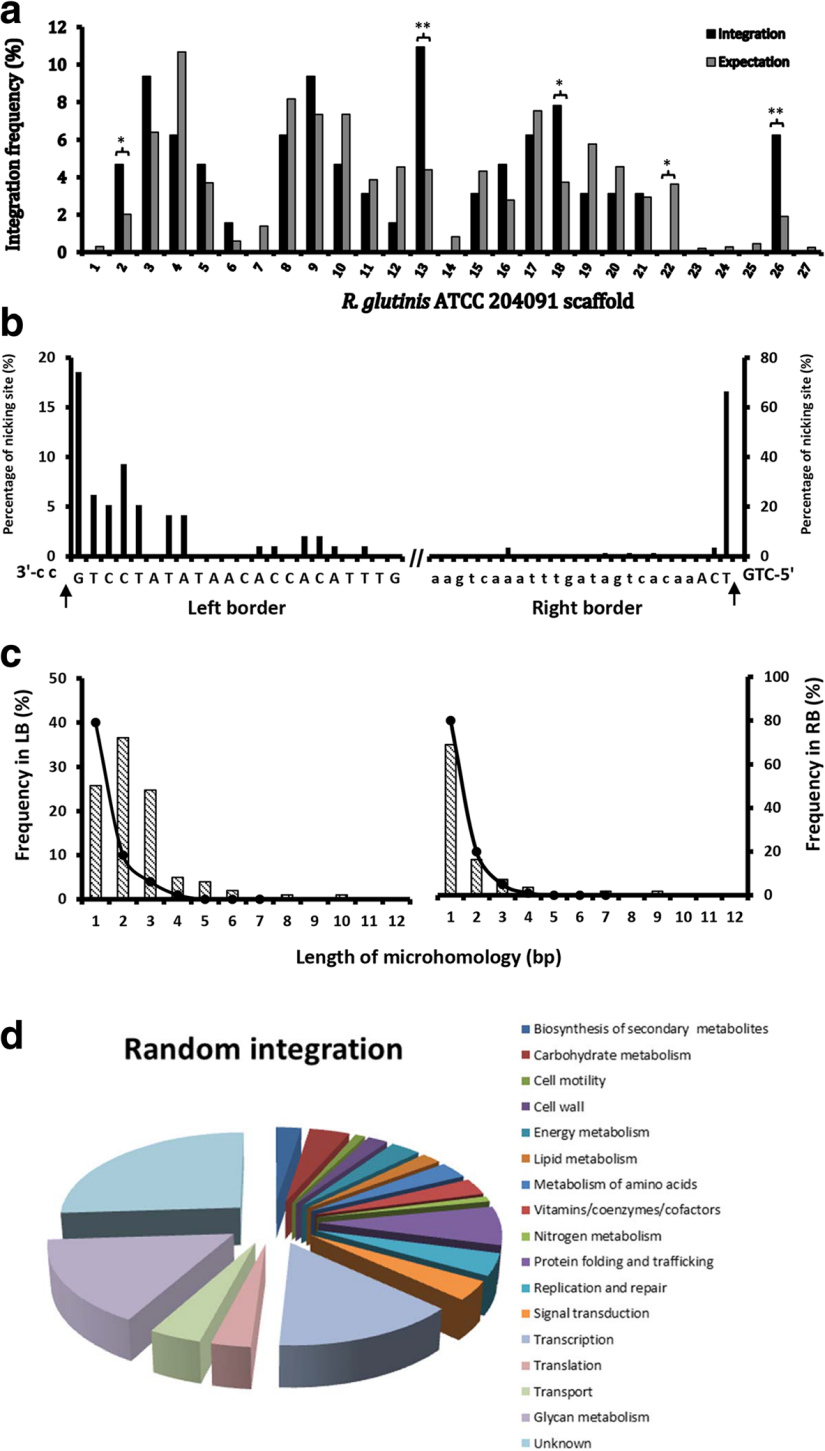
Identification of T-DNA tagging sites. **a** Genomic distribution of T-DNAs. hiTAIL PCR product sequences were searched against the genome database of *R. glutinis* ATCC 204091. **b** Nicking positions of the integrated T-DNAs. Histogram of nicking sites were presented based on the nicking frequency calculated from 61 LB and 196 RB flanking sequences. T-DNA border repeats were capitalized. Arrows indicate the positions of T-DNA nicking by *Agrobacterium*. **c** Size distribution of microhomology. Columns show the distribution of microhomology sizes found in this study. Black line indicated the expected sizes of microhomology based on calculation. **d** Classification of proteins affected by the 132 T-DNA tags

The genome sequences adjacent to T-DNA tagging positions were analyzed by high efficiency thermal asymmetric interlaced PCR (hiTAIL PCR) [37, 38]. A total of 480 samples were analyzed, including 192 transformants of *R. toruloides* ATCC 10657 analyzed for both left border (LB) and right border (RB) flanking sequences, and 96 transformants of *R. glutinis* ATCC 90781 for LB flanking sequences only. The success rate of HiTAIL PCR was 72.5% (346/480), which yielded 268 high-quality sequencing results (77.5%). To identify the chromosomal positions of T-DNA insertions, 61 LB flanking sequences were analyzed by BLASTn searches against the *R. glutinis* ATCC 204091 genome database (Additional file 2: Table S1). As expected, T-DNAs were mapped to the majority of scaffolds (21 out of 29), in which scaffold No. 2, 13, 18 and 26 showed the highest number of hits (Fig. 4a). Scaffolds that missed the analysis were all small in size.

T-DNA integration is known to be initiated at the RB, with DNA nicks generated between the 3rd and 4th nucleotide of the 25 bp border repeat sequence (RB canonical insertion). Generally, deletions of various lengths are generated in the RB region of inserted T-DNAs [47]. Our results showed that T-DNA integration exhibited much higher accuracy at RB than LB (Fig. 4b), with 30.8% (79 of 172) of the inserted T-DNAs containing deletions (ranging from 1 to 80 bp) at RB end compared with 79.1% deletions (68 of 86) at the LB end.

Microhomology at the insertion junctions was also examined. 73.7% (101 of 137) of LB insertions showed homology of 4 bp or more, compared with 42.0% (55 of 131) at RB. Microhomology of up to 10 bp was found although it was usually less than 4 bp (Fig. 4c). Collectively, T-DNA integration in *Rhodotorula* species does not require long stretch of sequence homology at the cross-over position. This is similar to the illegitimate recombination reports in other species, such as budding yeast *Saccharomyces cerevisiae* [48], corn smut fungi *Ustilago maydis* [39], rice blast fungi *Magnaporthe oryzae* [49], plants [50] and mammals [51].

The DNA sequences within 1.0 kb from the insertion sites were annotated. As shown in Additional file 3: Table S2, most mutants could be functionally assigned: 15.8% are likely to be involved in glucan metabolism, 14.3% in transcription, 7.1% in protein folding and trafficking, 4.1% in carbohydrate metabolism, 4.1% in DNA replication and repair, and 4.1% in transport (Fig. 4d). The rest of the mutants (26.0%) could not be functionally assigned (presented as “Unknown” in Fig. 4d). It was obvious that T-DNA insertions had a strong bias towards gene coding and regulatory regions (Table 1 and Additional file 3: Table S2). Taken together, T-DNA insertions could be exploited to tag a wide range of genes.

**Table 1:** Distrubution of T-DNA insertion positions

Locations	Tagged loci	Percentage (%)
Upstream 0.5–1.0 kb	13	10.7
Upstream 0.5 kb	18	14.9
Downstream 0.3 kb	5	4.1
Coding sequence	75	62.0
Intergenic sequence	10	8.3
Total	121	100.0

### Direct identification of lipogenic mutants

Cerulenin, (2S)(3R)2,3-epoxy-4-oxo-7,10-dodecadienoylamide, was discovered from the culture broth of *Cephalosporium caerulens* [52, 53]. It has been used as a fungicide due to the inhibition effects on the biosynthesis of fatty acids and steroids [54]. Mutants that survive cerulenin treatment are expected to produce higher levels of lipid or polyunsaturated fatty acids (PUFAs), and the relevant genes would be useful for lipid metabolic engineering [55–57]. Approximately 10,000 transformants were screened against 50 μg/mL cerulenin in the selection plates, a level that fully blocked the growth of wild-type (WT) cells (Additional file 4: Figure S2A). A total of 12 T-DNA tagged strains that suvvived the treatment were collected and termed as *Rhodotorula* Cerulenin Mutants, RCM1 to RCM12. Notably, the average α-linolenic acid (ALA, C18:3Δ^9,12,15^) level, an omega-3 PUFA naturally produced in *R. toruloides*, was significantly improved in the 12-member mutant population (Fig. 6a). In particular, RCM6 produced ~ 3 folds higher level of ALA than WT (Fig. 6a).

Secondly, Nile red, a fluorescent dye used extensively for intracellular lipid tracking [58] and rapid estimation of intracellular lipid content [59], was tested as a selection marker. A T-DNA mutant library with ~ 10,000 transformants was selected on nile red-containing YPD agar plates. Visual examination under a fluorescent dissecting microscope (Additional file 4: Figure S2B) yielded 4 mutants exhibiting stronger fluorescence intensities. The mutants were named *Rhodotorula* Nile red Mutants, RNM1 to RNM4. RNMs had significantly higher lipid contents than WT although they showed little differences in fatty acid profiles (Fig. 6b). Interestingly, RNM1 had its T-DNA inserted into the 3’ UTR region of a putative omega-3 fatty acid desaturase gene (Table 2), resulting in a 47% increase in lipid accumulation (Fig. 6b).

**Table 2:** T-DNA insertion sites of various mutants

Sequence code^*a*^	Genic site^*b,c*^	Best hit^*d*^	Annotation^*e*^	*Organism* ^*f*^	Identity^*g*^
RB sequences					
RCM1	Upstream-0.5 kb	XP_001549261	cellulose-binding GDSL lipase/acylhydrolase	*Botryotinia fuckeliana*	26%
RCM2	Upstream-1.0 kb	GENE ID: 5,545,759 Kpol_534p16	mannosyltransferase	*Vanderwaltozyma polyspora*	77%
RCM3	Upstream-0.5 kb	XP_001789963	regulator of nonsense transcripts; NADP-dependent isocitrate dehydrogenase	*Bos taurus*	40%
RCM4	Genic sequence	XP_001607008.2	protein bric-a-brac 2 isoform X1	*Nasonia vitripennis*	29%
RCM5	Upstream-0.5 kb	XP_003174510	C6 zinc finger domain-containing protein	*Arthroderma gypseum*	32%
RCM6	Genic sequence	XP_001629556	Fatty aldehyde dehydrogenase	*Nematostella vectensis*	51%
RCM7	Genic sequence	XP_571856	Hexose transport-related protein	*Cryptococcus neoformans*	53%
RCM8	Genic sequence	XP_001731990	Transcription initiation factor TFIID subunit 2	*Malassezia globosa*	40%
RCM9	Genic sequence	NP_001125572	stAR-related lipid transfer protein 3	*Pongo abelii*	29%
RCM10	Upstream-1.0 kb	XP_001645395	GPI mannosyltransferase 3	*Vanderwaltozyma polyspora*	77%
RCM11	Upstream-0.5 kb	XP_662119	Cell wall protein that functions in the transfer of chitin to beta (1–6) glucan	*Aspergillus nidulans*	36%
RCM12	NA^*h*^	–	–	*–*	–
RNM1	Downstream 0.3 kb	AAC98967.2	omega-3 fatty acid desaturase	*Vernicia fordii*	41%
RNM2	NA				
RNM3	Upstream 0.5 kb	ZP_03104366	amino acid permease	*Bacillus cereus W*	87%
RNM4	NA				
RTM1	Genic sequence	XP_016273537	SH3 domain-containing protein	*Rhodotorula glutinis NP11*	93%
RTM2	Upstream 0.5 kb	EGU13095.1	salicylate hydroxylase	*Rhodotorula glutinis ATCC 204091*	73%
RTM3	Genic sequence	XP_501740.1	nitrogen assimilation transcription factor	*Yarrowia lipolytica*	71%
RTM4	Upstream 0.5 kb	ZP_08453184.1	putative zinc-binding oxidoreductase	*Streptomyces sp.*	47%
RTM5	Genic sequence	ZP_07628725.1	putative lipoprotein	*Prevotella amnii*	45%
RTM6	Genic sequence	YP_001220603.1	resolvase site-specific recombinase	*Aeromonas bestiarum*	94%
RAM1	Genic sequence	XP_003032296	Riboflavin transporter MCH5	*Schizophyllum commune*	52%
RAM2	Upstream-0.5 kb	YP_001220603	resolvase	*Aeromonas bestiarum*	95%
RAM3	Genic sequence	XP_571856	hexose transport-related protein	*Cryptococcus neoformans*	36%
RAM4	Genic sequence	XP_758766	TATA-binding protein associated factor	*Ustilago maydis*	35%
RAM5	Genic sequence	AHB14354	phytoene synthase	*Rhodosporidium diobovatum*	98%

^***a***^Sequence codes are given according to the mutant names

^***b***^T-DNA tagged genes were determined according to the BLASTx results

^***c***^“Upstream 1.0 kb”, “Upstream 0.5 kb” and “Downstream 0.3 kb” denotes T-DNA inserted 501–1000 bp, 1–500 bp upstream of the putative translation initiation codon (ATG) and 1–300 bp downstream from the putative stop codon, respectively

^***d***^Best hit denotes the BLASTx result with the highest E-score

^***e***^Annotations were determined according to the BLASTx results

^***f***^Microorganism denotes the host of Best hit

^***g***^Identity values were from BLASTx results

^***h***^Not available due to poor sequencing result

Thirdly, tetrazolium violet, a redox indicator that gives colonies a distinct violet color if the cells accumulate lipids [60, 61], was tested as a selection marker for lipogenic mutants. As expected, supplementation of 10 μg/mL of tetrazolium violet in selection medium resulted in pigmented transformants (Additional file 4: Figure S2C). Screening of ~ 3000 transformants yielded 6 mutants with deeper pigmentation. The strains were named *Rhodotorula* Tetrazolium violet Mutants, RTM1 to RTM6. Again, RTMs had higher lipid content than WT (Fig. 6c), with little changes in the fatty acid compositions (data not shown).

The T-DNA insertion sites of above 22 mutants were analyzed by hiTAIL PCR, and 19 T-DNA flanking sequences were successfully identified (Table 2). The distribution of T-DNA insertion sites appeared similar to previous results (Table 1), where the T-DNAs were integrated in gene coding regions or regulatory regions within 1.0 kb upstream to the 0.3 kb downstream of the coding sequence (Table 2). The affected gene products of RCMs showed high correlation with the lipogenic bioprocess (Table 2). Similarily, the affected gene products of RNMs and RTMs were predicted to be involved in the metabolism of lipids (RNM1, RTM5), amino acids (RNM3), energy (RTM2), signal transduction (RTM1) and transcription (RTM3, 4 and 6).

### Direct identification of carotenoid production mutants

**Fig. 5:**
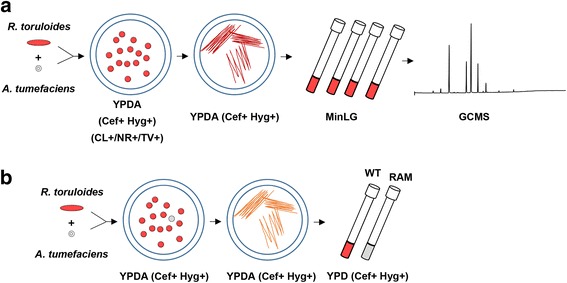
Schematic diagram for gene identification based on IM. **a** Screening for lipid/fatty acid production mutants. **b** Visual screening for carotenoid production mutants. Abbreviations: CL+, cerulenin (50 μg/mL); NR+, nile red (0.5 μg/mL); TV+, tetrazolium violet (10 μg/mL); Cef+, cefotaxime (300 μg/mL); Hyg+, hygromycin B (150 μg/mL)

To discover novel genes that are involved in the regulation of carotenoid biosynthesis, we designed a simple screening strategy based on the changes of colony color (Fig. 5b). From a population of ~ 20,000 T-DNA tagged mutants, 1 yellowish and 4 albino transformants were found and named *Rhodotorula* Albino Mutants, RAM1 to RAM5 (Fig. 7a and Additional file 4: Figure S2D). Sequence analysis revealed that T-DNA was inserted into the DNA sequence encoding a putative riboflavin transporter, resolvase, hexose transporter, TATA-binding protein associated factor and phytoene desaturase, respectively (Table 2). These data suggest that new factors for carotenoid biosynthesis could be identified through the IM approach.

### Validation of RAM5

To validate the gene tagging strategies used, the albino mutant RAM5 was analyzed in detail (Fig. 7a). BLAST search of the hiTAIL PCR product showed that the T-DNA was inserted between nucleotide 391,802 and 391,803 in scaffold No.18 (AEVR02000018), disrupting the putative phytoene desaturase gene (*CAR1*, genome locus RTG_00274) at the 3rd exon. To validate the result, *CAR1* was deleted in WT through homologous recombination, which led to the replacement of the genome sequence between + 948 and + 2097 (from the translational start of *CAR1*) by the hygromycin resistance gene cassette (P_*GPD1*_*::hpt-3::*T*nos*, Fig. 7b). Indeed, the resulting *car1Δ* mutant, which was confirmed by Southern blot analysis (Fig. 7c), showed similar creamy color as the T-DNA tagged mutant, RAM5 (Fig. 7d). Furthermore, re-introduction of the wild-type *CAR1* sequence (− 662 to + 2928, Fig. 7b) into the genome of *car1Δ* (the resulting mutant *car1C*) fully restored the cell color (Fig. 7d). HPLC analysis of carotenoids showed that the main carotenoid species produced in WT *R. toruloides*, such as torulene, torularhodin, γ-carotene and β-carotene [62–64], were totally absent in *car1Δ*, and fully restored in *car1C* (Fig. 7e and f). qRT-PCR analysis confirmed that the transcripts of *CAR1* were undetectable in *car1Δ* and restored in *car1C* (Fig. 7g). These data confirmed that *CAR1* encodes a key enzyme in carotenoid biosynthesis. Thus, the successful identification of *CAR1* further demonstrates that gene identification and strain improvement strategy based on T-DNA insertional mutagenesis is effective and reliable.

## Discussion

*R. toruloides* is an unusual yeast species with highly efficient oil and carotenoid production capacity. However, its potential as an industrial host remains largely unexploited, in part because of the lag in the development of genetic tools. In this study, we report comprehensive studies on factors affecting ATMT efficiency. The data complements our previous report on the transformation method for this yeast [16]. To our surprise, some conditions such as the choice of membrane, pH value and agar concentration used for co-culture, were critical for the TFE. Even a minor change of the co-culture pH could be fatal for transformation (Fig. 2a). Therefore, it is advisable to optimize medium pH, co-culture time and donor/recipient ratio when a new strain or taxa of yeast is used for ATMT. As the Um*gpd*::*hpt-3* selection cassette has also been used successfully in the transformation of *U. maydis* and *U. scitaminea* [16, 65], it should be broadly useful for dominant selection in both *Ustilaginomycotina* and *Puicciniomycotina* subphyla.

In addition, it was feasible to perform ATMT without the use of supporting membrane for co-culture. The transformed cells could be transferred to selection plates by spreading (as was used herein), “wash and plate”, replica printing or medium over-lay. Avoiding the use of membranes could also be appealing to researchers in under-developed countries.

**Fig. 6:**
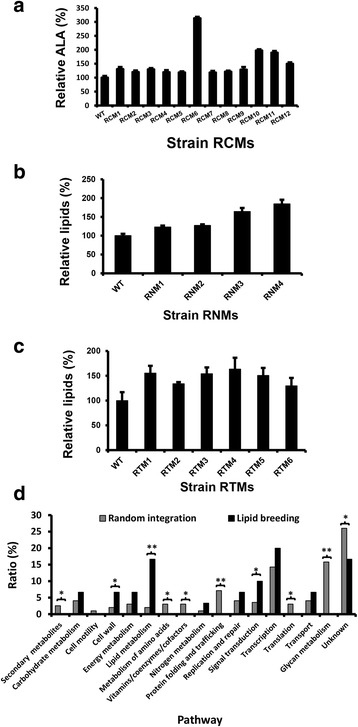
Characterization of lipogenic mutants. **a** Relative α-Linolenic acid (C18:3Δ^9,12,15^, ALA) yields of RCM mutants. **b** Relative lipid contents of RNM mutants. **c** Relative lipid contents of RTM mutants. **d** Mutant classification and statistical analysis (Chi-square). 22 lipogenic mutants (RCMs, RNMs and RTMs) were plotted according to their predicted protein functions (Dark bars). The predicted percentage of protein fucntions affected were shown in grey bars. Probability: *, *P* < 0.05; **, *P* < 0.01

Four screening strategies have been tested for direct identification of genes of interest, each with a specific focus. It is encouraging that mutants could be identified in all cases, leading to the discovery of 27 mutants in total. Importantly, many of the genes appeared to be consistent with their expected roles. For example, riboflavin transporter is involved in the uptake of riboflavin (vitamin B2) and flavin adenine dinucleotide (FAD), which are co-factors for many biocatalytic reactions [66]. Hexose transporter is involved in the uptake of monosaccharides, which is regarded as the first and rate-limiting step of glucose metabolism [67]. Most notably, disruption of the putative aldehyde dehydrogenase gene can lead to a significant increase in ALA content (see RCM6 in Fig. 6a). As a proof of concept, one of the mutants (RAM5) was validated by reverse genetics and the results were in full agreement with the prediction that the gene was involved in the biosynthesis of carotenoids. The latter has been confirmed by another laboratory recently [68]. A full characterization of the 27 mutants is expected to yield valuable information on novel strategies to improve lipid and carotenoid production in this yeast.

Single-copy integration rate, the percentage of transformants with a single copy of transgene integrated into the genome, is an important parameter for IM studies, which is particularly relevant for gene tagging work. The non-homologous end-joining (NHEJ) DNA recombination pathway is believed to be essential for ATMT [69]. Previously, ATMT of *U. maydis* using the same method yielded a single-copy integration rate of 96% [39]. A significantly lower single-copy integration rate of 75% was observed in *R. toruloides*. This could be attributed to the weaker activity of the *U. maydis gpd1* promoter that was used to drive the expression of hygromycin resistance gene in *R. toruloides* [16]. Indeed, single-copy integration rate was almost 100% when it was replaced with the endogenous Rt*GPD1* promoter (our unpublished data). The average T-DNA copy number (1.36) in *Rhodotorula* species remained lower than in plants, such as 1.5 in *Arabidopsis* [47] and 1.76–2.0 in rice [70, 71]. On the other hand, multiple-copy T-DNA integrations could result in the higher expression of target protein, which could be exploited for metabolic engineering in this yeast.

## Conclusions

We have established a trackable and reliable mutagenesis method for *R. toruloides* using T-DNA as the mutagen. This method will be valuable for gene discovery as well as strain improvement in *Pucciniomycotina* subphylum and beyond. The 27 mutants identified in this study should yield significant novel information on the lipid and carotenoid biosynthetic pathways.

## Methods

### Strains, chemicals, media and culture conditions

*R. toruloides* strains ATCC 10657, ATCC 10788, ATCC 204091, *R. glutinis* strain ATCC 90781, and *A. tumefaciens* strain AGL1 [72] were obtained from ATCC (USA). *R. graminis* strain WP1 and *Sporobolomyces roseus* strain FGSC 10293 (IAM13481) were obtained from Fungal Genetics Stock Center, University of Missouri, USA. *R. toruloides KU70* null mutant strain ku70e was described previously [26].

Hygromycin B was purchased from Roche Diagnostics (USA). Nylon N and N+ membranes (Φ 82 mm, 0.45 μm) were from GE Healthcare (Uppsala, Sweden), cellulose acetate membrane (47 mm, Φ0.45 μm) from Grace (Deerfield, IL, USA), cellulose nitrate (87 mm, Φ0.45 μm) from Schleicher & Schuell (Dassel, Germany) and filter paper (Grade 4, Φ90 mm, 20–25 μm in thickness) from Whatman (USA). Cerulenin was obtained from Sigma-Aldrich (USA) and prepared as a 5 mg/mL stock in DMSO. All other chemicals were also obtained from Sigma-Aldrich.

*Rhodotorula* strains were cultured at 28 °C in YPD broth (10 g/L yeast extract, 20 g/L peptone, 20 g/L glucose) or on solid potato-dextrose agar (PDA). *A. tumefaciens* was grown at 28 °C in either liquid or solid 2YT medium (16 g/L tryptone, 10 g/L yeast extract, 5 g/L NaCl). Carotenoid production medium B2001 was prepared as described previously [73]. It contained (per litre) 46 g glucose, 11.74 g yeast extract, 2 g K_2_HPO_4_, 2 g KH2PO_4_, 0.1 g MgSO_4_·7H_2_O, 18 g threonine, 10 mL trace element (TE) solution, pH 6.0. TE solution (per litre) contained 4.0 g CaCl_2_·2H_2_O, 0.55 g FeSO_4_·7H_2_O, 0.52 g citric acid·H_2_O, 0.1 g ZnSO_4_·7H_2_O, 0.076 g MnSO_4_·H_2_O and 0.1 mL smoked H_2_SO_4_ [74]. Lipid production medium MinLG was prepared as previously described [75] with some modification. It contained (per litre) 30 g glucose, 1.5 g yeast extract, 0.5 g (NH_4_)_2_SO_4_, 2.05 g K_2_HPO_4_, 1.45 g KH_2_PO_4_, 0.6 g MgSO_4_·7H_2_O, 0.3 g NaCl, 10 mg CaCl_2_, 1 mg FeSO_4_, 0.5 mg ZnSO_4_, 0.5 mg CuSO_4_, 0.5 mg H_3_BO_4_, 0.5 mg MnSO_4_ and 0.5 mg NaMoO_4_, pH 6.1. A seed culture in YPD broth was inoculated in medium B2001 and MinLG to initiate the production of carotenoids and lipids, respectively. Unless indicated otherwise, either lipid or carotenoid production was conducted at 28 °C for 4 days with constant agitation (250 rpm).

### DNA constructs

Oligonucleotides used are listed in Additional file 5: Table S3. All restriction and modification enzymes were from New England Biolabs (NEB, Massachusetts, USA). Binary vectors pEX2 [16] are pPZP200 derivatives used for dominant selection against hygromycin B.

The promoter of *Ashbya gossypii* translational elongation factor 1-α gene (P_*tef*_, 348 bp) [76], *Ustilago maydis gpd1* (P_*gpd*_, 595 bp in length) [39, 77], *Aspergillus nidulans gpdA* (P_*gpdA*_, 884 bp) [78] and *R. toruloides GPD1* (P_*GPD1*_, 1429 bp) [16], were amplified from plasmid pTHPR1 [39], genomic DNA of *U. maydis*, *A. nidulans* and *R. toruloides*, respectively. The primer pair used for the amplication of P_*gpd*_, P_*gpdA*_, P_*tef*_ and P_*GPD1*_ was Pgap-Sf/Pgap-Nr, PgpdA-Sf/PgpdA-Nr, Ptef-Sf/Ptef-Nr and Rt011S/Rt012N, respectively. The resulting DNA fragment of P_*gpd*_, P_*gpdA*_, P_*tef*_ and P_*GPD1*_ was double-digested with SpeI and NcoI and used in a 3-fragment ligation with the 1030-bp BspHI/SmaI DNA fragment of the synthetic *hpt-3* gene casette [16] and the 8855-bp SpeI/SacI (blunt-ended) DNA fragment of pEC3GPD-GUS (Additional file 6: Figure S3A) to create pEC3UmGPD-HPT3, pEC3GPDA-HPT3, pEC3TEFA-HPT3 and pEC3GPD1-HPT3, respectively (Additional file 6: Figure S3B).

To delete *CAR1*, the genome sequence ranging from − 89 to + 2928 from the translational start of *CAR1* (AEVR02000018) was amplified using oligos Rt127–2 and Rt128–2. The resultant blunt-ended PCR product was ligated to PmeI and SacI (blunt-end) digested pEX2 to create the intemediate vector pEX2CAR1. The partial coding sequence of *CAR1* in pEX2CAR1 was digested by SacII and MfeI (both blunt-ended) and replaced with the hygromycin resistance cassette P_*GPD1*_*::hpt-3::*T*nos* [26] to create the gene deletion plasmid pKOCAR1. To make the complementation plasmid pRHCAR1C, the genomic sequence of *CAR1* ranging from − 662 to + 2928 from the translational start was amplified using oligos Rt319Sf and Rt128–2, 5′-hydroxyl termini phosphorylated with T4 polynucleotide kinase, digested with SpeI, and inserted to the SpeI and EcoRV sites of pRH201.

### Nucleic acid preparations and manipulations

Genomic DNA was isolated using MasterPure Yeast DNA Purification Kit (Epicentre Biotechnologies, Madison, WI, USA). Total RNA was prepared using the RiboPure RNA Purification Kit (ThermoFisher Scientific, Austin, TX, USA). The resulting nucleotide acids were qualified and quantified by agarose gel electrophoresis and NanoDrop® ND-1000 Spectrophotometer (Nanodrop Technologies, USA), respectively.

### *Agrobacterium tumefaciens*-mediated transformation

Fungi transformation via ATMT was performed as described previously unless indicated otherwise [16]. For membrane-free ATMT, *Agrobacterium* and fungi cells were mixed and spread on the surface of IM agar [16] without any supporting membranes. After co-culture at 24 °C for 2 days, cells were scrapped out using a L-shape spreader and plated on the surface of YPD agar supplemented with appropriate antibiotics as described previously [16], and incubated at 30 °C until the appearance of transformants.

### Southern blot analysis

**Fig. 7:**
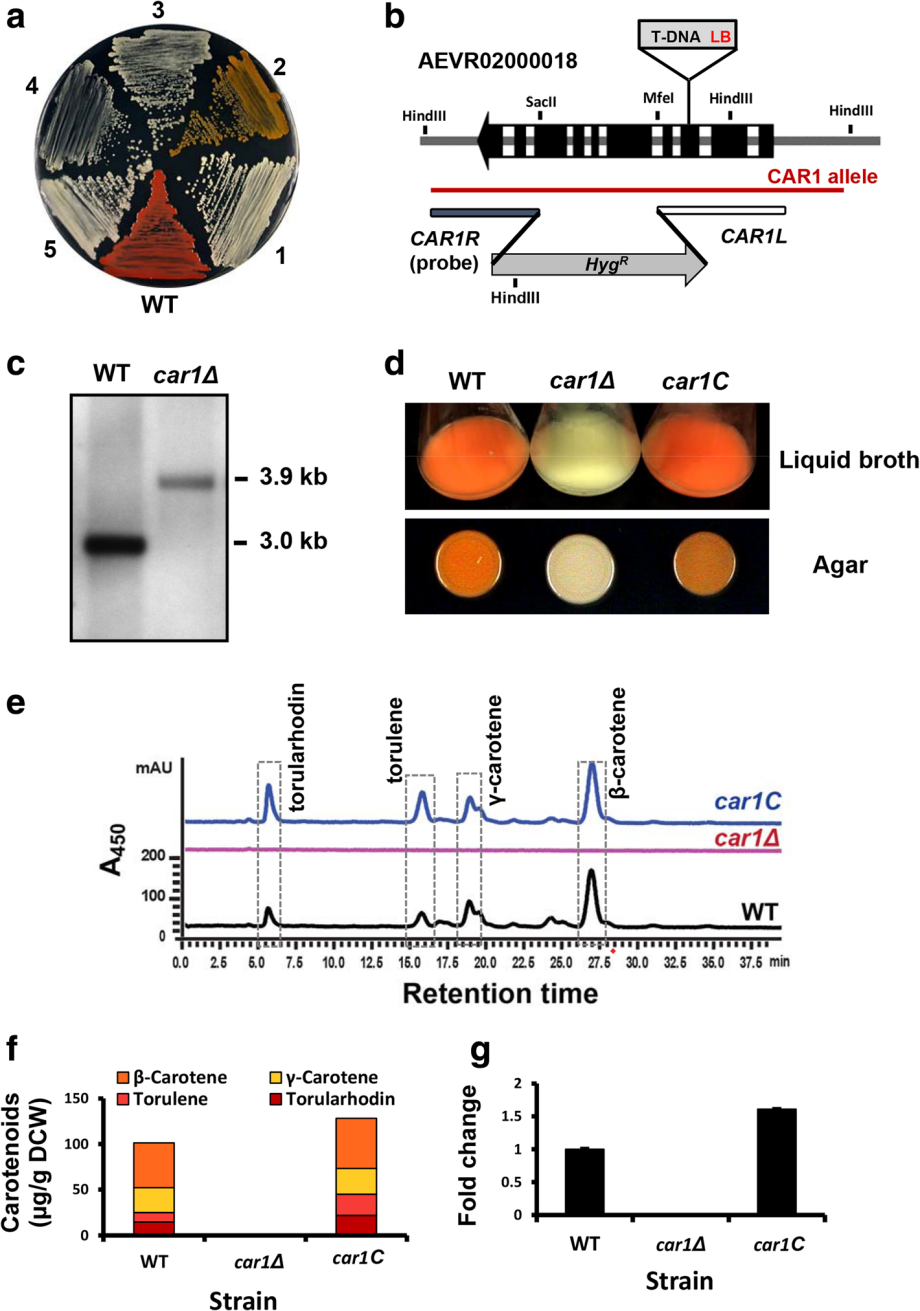
Identification of carotenogenic mutants and functional validation of RAM5. **a** Colony color phenotypes of RAMs. All strains were streaked on PDA plate and incubated at 28 °C for 2 days. **b** Schematic diagram of *CAR1* structure and its deletion and complementation strategies. *CAR1* genomic sequence (Dark red line) ranging from − 662 to + 2928 was used to complement the *car1* mutant. **c** Southern blot hybridization of candidate *CAR1* null mutant (*car1Δ*). **d** Pigment colors of WT, null mutant (*car1Δ*) and complementation strain (*car1C*) in either liquid broth or agar medium. **e** HPLC chromatogram of carotenoids from WT, *car1Δ* and *car1C*. All strains were cultured in medium B2001 for 5 days. **f** Quantitative analysis of 4 different carotenoid species (microgram of carotenoid per dry cell weight, μg/g DCW) in WT, *car1Δ* and *car1C* strains. **g** Changes of *CAR1* mRNA levels in WT, *car1Δ*, and *car1C*. All cells were cultured in carotenoid production media B2001 for 1 day before RNA extraction. Actin encoding gene (*ACT1*) was used as the reference

Genomic DNA was digested with PstI and separated by electrophoresis on 0.8% agarose gels. DIG-labeled probe of the partial *hpt-3* gene (+ 375 through + 1036) was amplified using oligos HptRU and HptRSL2 (Additional file 5: Table S3). To verify *CAR1* deletion mutants, genomic DNA was digested with HindIII and the digoxigenin-labeled *CAR1R* fragment (Fig. 7b) was used as the probe. Southern hybridization was carried out according to the manufacture’s instructions (DIG-High prime DNA labeling and detection starter Kit II, Roche Diagnostics).

### Identification of T-DNA tagging positions

T-DNA tagging positions in the genome were identified using hiTAIL PCR. Specific primers (HRSP1, HRSP2 and HRSP3) and arbitrary primers (LAD1–1 and LAD1–4) were used for LB flanking sequences. Specific primers (HRRSP1, HRRSP2 and HRRSP3) and arbitrary primers (LAD1–1 and LAD1–4) for RB flanking sequences. PCR were carried out with i-Taq DNA polymerase (i-DNA Biotech, Singapore) in a PTC-200™ Programmable Thermal Controller (Bio-Rad, USA). PCR products were purified using gel extraction kit (Qiagen, CA, USA) and sequenced using the Big Dye v3.1 terminator kit (Applied Biosystems, USA) with oligo HRSP3 (for LB) or HRRSP3 (for RB). In some cases, PCR products were cloned into pGEM-T easy vector (Promega, USA) by TA cloning technique and sequenced using oligos M13FP and M13RP.

### Quantitative reverse transcription PCR (qRT-PCR)

qRT-PCR was performed in triplicates as described [21]. Relative gene expression levels were calculated against the reference gene *ACT1* (actin encoding gene, GenBank acc. no. KR138696) [17] using the 2^-ΔΔCt^ method (RQ Manager software v1.2.1, Applied Biosystems). Oligonucleotide pair used for *CAR1* and *ACT1* was qCAR1f/qCAR1r and qACT1f/qACT1r, respectively.

### Screening for lipid and carotenoid production mutants

The genome of *R. toruloides* was mutagenized by random insertion of T-DNA of plasmid pRH201. Candidate lipid production mutants were selected by supplementation of various chemicals, such as 50 μg/mL cerulenin, 0.5 μg/mL nile red or 10 μg/mL tetrazolium violet. After incubation at 28 °C for 5 days, transformants that survived cerulenin treatment (Additional file 4: Figure S2A), showing higher fluorescent intensity on nile red-containing media or darker purple-color pigmentation (Additional file 4: Figure S2B) on tetrazolium violet-containing media (Additional file 4: Figure S2C), were transferred to YPD broth (300 μg/mL cefotaxime and 150 μg/mL hygromycin) for propagation and cryopreservation. Candidate carotenoid production mutants were selected by visual screening of transformants.

### Extraction of lipids and carotenoids

Total lipid was extracted essentially as described previously [79]. Dry cell biomass (10 mg) was mixed with 500 μl of 4 M HCl and incubated in a boiling water bath for 15 min. Subsequently, samples were placed in a -20 °C freezer for at least 1 h and cell lysates were mixed with 1.0 mg pentadecanoic acid (C15:0, the internal standard for the subsequent GC analysis) and 1.0 mL chloroform:methanol (2:1, *v*/v). After centrifugation, the lower solvent phase was transferred to a new tube. The total lipid mass was determined by weighing after drying in a vacuum concentrator (Eppendorf, USA).

Extraction of carotenoids was performed as described previously [80]. Birefly, cells from 50 mL cultures were pelleted by centrifugation and washed twice with water. Equal amount of acid-washed glass beads (0.4–0.6 mm in dimeter, Sigma-Aldirch) and 5 mL DMSO were added and mixed vigorously for 10 min by vortexing. After incubated at 65 °C for 1 h, freezed at -20 °C for 30 min and centrifuged, the supernatants were removed to a new tube (DMSO-dissolved carotenoids). The pellets were mixed with 30 mL of light petrolium ether-ethyl acetate (2:1, *v*/v) for 10 min by vigorous vortexing. After centrifugation, the supernatants (solvent-dissolved carotenoids) were combined with previous DMSO-dissolved carotenoids and then 2 mL of saturated NaCl solution was added. The upper solvent phase was separated and blow-dried with nitrogen gas and carotenoids was re-dissolved in hexane.

### Quantification methods

Cell biomass (dry cell weight) was determined by lyophilizing the cell pellet until constant weight was reached.

Residual glucose was quantified by HPLC in a Prominence ultra fast liquid chromatography (UFLC) system (Shimadzu, Kyoto, Japan). Culture was filtered through a 0.2 μm nylon membrane and run through a 300 × 7.0 mm Aminex 87H column (Bio-Rad) at a constant flow rate of 0.7 mL/min using 5 mM sulfuric acid as the mobile phase. The column was maintained at 50 °C and glucose was detected with a Refractive Index Detector (RID, Shimadzu). Concentration of glucose in the cell culture was determined using calibration curves built with the standard glucose aqueous solution.

Quick estimation of lipid content was performed as described previously [59]. Briefly, 10 μl cell culture and 2 μl nile red stock (50 mM in acetone) were mixed with 200 μl PBS buffer (pH 7.4) in a well of a FluoroNunc plate (Thermo Fisher Scientific, Langenselbold, Germany). Each sample was accompanied with a nile red-free well as the background control. Another fraction of the cell culture (10 μl) was mixed with 90 μl PBS buffer (pH 7.4) in a 96-well flat-bottom transparent plate (Nunc, Roskilde, Denmark) to measure cell optical density. The data was acquired and analyzed using the Infinite M200 plate reader and the iControl™ version 3.0 software (Tecan, Salzburg, Austria). Cell optical density was read at 600 nm after subtracting background while fluorescence intensity was measured with an excitation and emission wavelength of 488 nm and 508 nm, respectively. The relative lipid content was calculated by normalization against its absorptance at 600 nm after deducting the background control. Statistical triplicates were used for all tests.

Fatty acid profiles were determined by gas chromatography-mass spectrometry (GCMS). Preparation of fatty acid methyl esters (FAMEs) and subsequent GCMS analysis were performed as described previously [81]. Briefly, lipids were dissolved in 300 μl petroleum ether-benzene (1:1, *v*/v), mixed with equal volume of methanolic hydrochloride acid (3 M, Sigma) and kept at 80 °C for 1 h. FAMEs were extracted with 1 mL of hexane, and 1 μL was injected to a HP-88 fused silica capillary column (30-m length, 0.25-μm diameter, and 0.25-mm film thickness, Agilent J&W Scientific, Folsom, CA, USA) fitted in a GCMS (QP2010 Ultra, Shimadzu). The running conditions were typically 42.3 mL/min nitrogen flow, 150 °C for starting temperature (3 min), a 15-min ramp to 240 °C, and holding at 240 °C for 7 min. The FAME peaks were identified by searching against Shimadzu NIST08 compound library and quantified as percentages of total fatty acids (%TFA).

The total carotenoid concentration was estimated by spectrophotometry method [82]. Briefly, the absorbance of carotenoids in petroleum ester was measured at 485 nm (*A*_*485*_) and the carotenoid concentration was calculated using the following formula: Carotenoids (mg/L) = *A*_*485*_ × 1000/2680, where the coefficient of absorbance used was that equivalent to β-carotene: E^1%^_1 cm_ = 2680 for petroleum ether. The carotenoid species were quantified by HPLC (Shimadzu Prominence UFLC system coupled with photodiode array detector) as previously described [83]. Briefly, carotenoids were filtered through a 0.2 μm nylon membrane and separated through the Kinetex C18 reverse phase column (100 × 3 mm, ɸ 2.6 μm, Phenomenex Inc., CA, USA) at a constant temperature of 35 °C. The mobile phase (acetonitrile:methanol containing 0.1 M ammonium acetate:dichloromethane = 71:22:7, v/v/v) was run at a constant flow rate of 0.3 mL/min. Various carotenoid compositions were quantified using β-carotene (C-4582, Sigma-Aldrich) as the external standard.

## Additional files

Additional file 1: **Figure S1.** Optimization of transformation conditions. Unless indicated otherwise, the same volume (100 μL) of *R. toruloides* strain ATCC 10657 and *A. tumefaciens* strain AGL1 harboring plasmid pRH201 were co-cultured on IM agar (pH 5.5 and Nylon N+ membrane) for two days, and subsequently selected on YPD agar medium (150 μg/mL hygromycin and 300 μg/mL cefotaxime) for 4 days. (A) The presence (+) and absence (−) of acetosyringone (100 μg/mL). (B) Co-culture time. (C) Volumetric ratio of fungi to *Agrobacteria*. 100 μL of fungal cells were co-cultured with 10 to 100 μL AGL1 (pRH201) on induction medium before selection. (D) Effect of various promoters for the expression of the synthetic *hpt-3* gene. *Um gpd1*, *Rt GPD1* and *An gpdA* represents the glyceraldehyde-3-phospohate dehydrogenase promoter of *U. maydis* (0.6 kb), *R. toruloides* (1.4 kb), and *Aspergillus nidulans* (0.8 kb), respectively. *Ag tef* represents the promoter of *Ashbya gossypii* translation elongation factor (245 bp). Transformation efficiency (TFE) was represented as the relative percentage value against the highest colony forming unit (CFU) observed in the trial. Biological triplicates were used and error bars represent the standard derivations. (PDF 85 kb)

Additional file 2: **Table S1.** Locations of 61 T-DNA left border flanking sequences. (PDF 91 kb)

Additional file 3: **Table S2.** Summary of 192 T-DNA flanking sequences in the *R. glutinis* ATCC 204091 genome. (PDF 159 kb)

Additional file 4: **Figure S2.** Chemical-assisted and visual screening systems. (A) Cerulenin-assisted screening for high PUFA producing mutants. - and + represents the absence and presence of 50 μg/mL cerulenin, respectively. (B) Nile red-assisted screening for high lipid producing mutants. L and H represents the low and high fluorescence intensity on 0.5 μg/mL nile red-containing YPD agar, respectively. (C) Tetrazeolium violet-assisted screening for high lipid producing mutants. L and H represents the low and high violet intensity on 10 μg/mL tetrazolium violet-containing YPD agar, respectively. (D) Visual screening for carotenoid producing mutants (indicated by arrow heads). (PDF 854 kb)

Additional file 5: **Table S3.** Sequences of oligonucleotides. (PDF 75 kb)

Additional file 6: **Figure S3.** T-DNA organizations in contructs used in this study. All binary vectors have the same pPZP200 backbone [84]. (A) pEC3Pxxx-HPT3. (B) pEC3GPD-GUS. LB: left border of T-DNA; RB: right border of T-DNA; P*xxx* represents three glyceraldehydes-3-phosphate dehydrogenase promoters from *A. nidulans* (P_*gpdA*_), *U. maydis* (P_*gpd*_) and *R. toruloides* (P_*GPD1*_) and the tranlation elongation factor promoter from *A. gossypii* (P_*tef*_). *hpt-3*: codon-optimized hygromycin resistance gene based on the codon usage bias in *R. toruloides*; GUS: *E. coli* β-glucuronidase gene; T_*35S*_: terminator of cauliflower mosaic virus 35S gene; Tnos: terminator of *A. tumefaciens* nopaline synthase gene; T_*tef*_: terminator of *A. gossypii* translation elongation factor gene; T_*cyc1*_: Terminator of *S. cerevisiae* iso-1-cytochrome C gene. The labeled restriction enzymes are unique cutting sites in the plasmid. (PDF 56 kb)

## Abbreviations

ATCC: American Type Culture Collection, USA
BLAST: Basic Local Alignment Search Tool (National Library of Medicine, National Institutes of Health, USA)
*CAR1*: Phytoene desaturase gene
DCW: Dry cell weight
*GPD1*: Glyceraldehyde 3-phosphate dehydrogenase gene
*hpt-3*: A synthetic *E. coli* hygromycin B phosphotransferase gene optimized according to the codon bias of *R. toruloides*
IM: Insertional mutagenesis
PUFA: Polyunsaturated fatty acid
RACE: Rapid amplification of cDNA ends
TFE: Transformation efficiency
UTR: Untranslated region
WT: Wild-type strain
