# Supplementary material for: Identification of novel genes in the carotenogenic and oleaginous yeast Rhodotorula toruloides through genome-wide insertional mutagenesis
Source: BMC Microbiol. 2018 Feb 21;18:14. doi: 10.1186/s12866-018-1151-6 (PMC5822628; doi:10.1186/s12866-018-1151-6)
Supplement: Supplementary file 1 — Figure S1. Optimization of transformation conditions. Unless indicated otherwise, the same volume (100 μL) of R. toruloides strain ATCC 10657 and A. tumefaciens strain AGL1 harboring plasmid pRH201 were co-cultured on IM agar (pH 5.5 and Nylon N+ membrane) for two days, and subsequently selected on YPD agar medium (150 μg/mL hygromycin and 300 μg/mL cefotaxime) for 4 days. (A) The presence (+) and absence (−) of acetosyringone (100 μg/mL). (B) Co-culture time. (C) Volumetric ratio of fungi to Agrobacteria. 100 μL of fungal cells were co-cultured with 10 to 100 μL AGL1 (pRH201) on induction medium before selection. (D) Effect of various promoters for the expression of the synthetic hpt-3 gene. Um gpd1, Rt GPD1 and An gpdA represents the glyceraldehyde-3-phospohate dehydrogenase promoter of U. maydis (0.6 kb), R. toruloides (1.4 kb), and Aspergillus nidulans (0.8 kb), respectively. Ag tef represents the promoter of Ashbya gossypii translation elongation factor (245 bp). Transformation efficiency (TFE) was represented as the relative percentage value against the highest colony forming unit (CFU) observed in the trial. Biological triplicates were used and error bars represent the standard derivations. (PDF 85 kb) [file 12866_2018_1151_MOESM1_ESM.pdf]

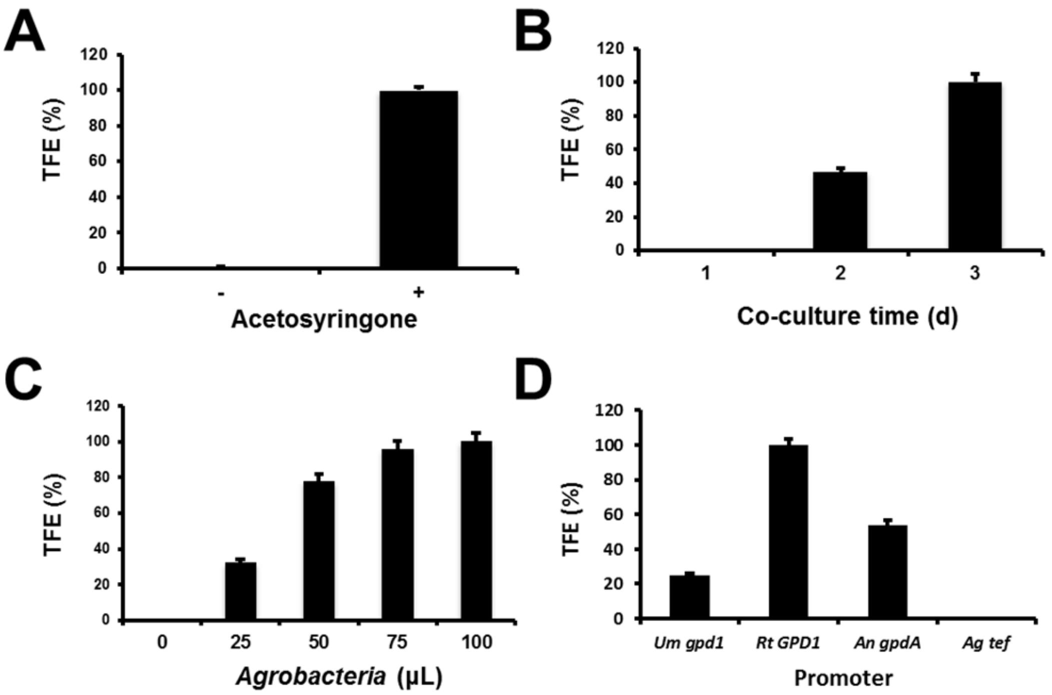

Additional file 1: Fig. S1. Optimization of transformation conditions. Unless indicated otherwise, the same volume (100 µL) of *R. toruloides* strain ATCC 10657 and *A. tumefaciens* strain AGL1 harboring plasmid pRH201 were co-cultured on IM agar (pH5.5 and Nylon N+ membrane) for two days, and subsequently selected on YPD agar medium (150 µg/mL hygromycin and 300 µg/mL cefotaxime) for 4 days. (A) The presence (+) and absence (-) of acetosyringone (100 µg/mL). (B) Co-culture time. (C) Volumetric ratio of fungi to *Agrobacteria*. 100 µL of fungal cells were co-cultured with 10 to 100 µL AGL1 (pRH201) on induction medium before selection. (D) Effect of various promoters for the expression of the synthetic *hpt-3* gene. *Um gpd1*, *Rt GPD1* and *An gpdA* represents the glyceraldehyde-3-phospohate dehydrogenase promoter of *U. maydis* (0.6 kb), *R. toruloides* (1.4 kb), and *Aspergillus nidulans* (0.8 kb), respectively. *Ag tef* represents the promoter of *Ashbya gossypii* translation elongation factor (245 bp). Transformation efficiency (TFE) was represented as the relative percentage value against the highest colony forming unit (CFU) observed in the trial. Biological triplicates were used and error bars represent the standard derivations.
