# Supplementary material for: Identification of novel genes in the carotenogenic and oleaginous yeast Rhodotorula toruloides through genome-wide insertional mutagenesis
Source: BMC Microbiol. 2018 Feb 21;18:14. doi: 10.1186/s12866-018-1151-6 (PMC5822628; doi:10.1186/s12866-018-1151-6)
Supplement: Supplementary file 2 — Table S1. Locations of 61 T-DNA left border flanking sequences. (PDF 91 kb) [file 12866_2018_1151_MOESM2_ESM.pdf]

Additional file 2: Table S1. Locations of 61 T-DNA left border flanking sequences[a]

Sequence number [a]	T-DNA Location [b] Scaffold	Position	Direction	Best hit [c]	Annotation [d]
A2	13	2105427	+	XP_016276022.1	phospholipid-translocating ATPase
A3	9	549003	-	XP_016273956.1	hypothetical protein
A4	12	734679	-	EGU12202.1	Succinate dehydrogenase
A5	23	1170864	-	XP_016270581.1	phosphoketolase
A6	2	603165	+		Integenic [e]
A7	12	735354	-	EGU12202.1	Succinate dehydrogenase
A8	12	456318	+	XP_016275283.1	methionyl-tRNA synthetase
A9	12	500284	-	XP_016275274.1	fungal cellulose binding domain containing protein
A11	28	354416	-	XP_016269482.1	citrate synthase
B1	28	354416	+	XP_016269482.1	citrate synthase
B7	12	734728	-	EGU12202.1	Succinate dehydrogenase
B8	11	59734	-	EGU13157.1	Nucleoporin
B9	26	321560	+		Integenic
B10	14	145647	+	XP_016272566.1	jumonji family transcription factor
B11	29	197513	-	XP_016276612.1	hypothetical protein
C2	8	519647	+	XP_016269747.1	GRF-type zinc finger protein
C3	23	67022	+	EGU13569.1	Proteophosphoglycan 5
C4	3	368994	-	EGU12700.1	Proteophosphoglycan ppg4
C5	17	630863	+		Integenic
C6	9	112655	-	EGU12202.1	Serine/threonine protein kinase FSK
C8	28	41929	+	EGU10835.1	Proteophosphoglycan ppg4
C10	3	1143363	+	XP_016274701.1	Chitin synthase 6
C12	10	646641	+	EGU11890.1	Proteophosphoglycan ppg4
D3	13	397604	-	EGU13218.1	DNA replication origin binding protein
D4	5	1009566	+	XP_016275084.1	nascent polypeptide-associated complex subunit alpha
D6	23	517672	+	EGU12270.1	Allergen
D7	12	735047	+	EGU12202.1	Succinate dehydrogenase
D8	10	17537	-	XP_016272680.1	F-box domain, cyclin-like domain containing protein
D9	7	198873	+	XP_016270676.1	C2H2-type zinc finger protein
D10	3	1143690	+	XP_016274701.1	Chitin synthase 6
D11	8	830880	-	XP_016271440.1	ABC transporter
D12	5	1009566	+	XP_016275084.1	nascent polypeptide-associated complex subunit alpha
E1	26	317846	+		Integenic
E3	13	1443808	-	XP_016275748.1	AP endonuclease 2
E4	14	471198	-	XP_016272433.1	hypothetical protein
E7	29	847216	-	EGU12439.1	Proteophosphoglycan ppg4
E8	12	735468	+	EGU12202.1	Succinate dehydrogenase
E9	3	1143245	+	XP_016274701.1	Chitin synthase 6
E11	3	1143768	+	XP_016274701.1	Chitin synthase 6
F1	23	999528	-		Integenic

F2	14	176340	+	XP_016272549.1	mitochondrial escape protein 2
F3	8	835911	+	EGU12878.1	putative DNA helicase INO80
F4	8	890709	+	EGU12891.1	Proteophosphoglycan ppg4
F6	17	680441	+		Integenic
F9	3	1142258	+	XP_016274701.1	Chitin synthase 6
F10	17	455168	+	JN208861.1	glyceraldehyde 3-phosphate dehydrogenase
F11	2	190370	+	XP_016272758.1	NCS2 allantoate transporter
G1	5	1001230	+	XP_016275079.1	WD40 repeat containing protein
G3	13	1765551	-	XP_016275884.1	Rho guanyl-nucleotide exchange factor
G4	1	704097	-	XP_016273496.1	SAM domain protein
G5	17	757299	-	XP_016271211.1	RNA recognition motif domain containing protein
G7	11	353553	+	XP_016274927.1	RNA-binding protein
G8	1	371526	-	XP_016273630.1	dimethyladenosine transferase
G12	25	464521	+	XP_016272965.1	Select seq ref|XP_016272965.1|
H2	23	817230	-	XP_016273025.1	hypothetical protein
H3	28	175370	-	XP_016270007.1	mRNA-decapping enzyme subunit 2
H5	25	464553	+		Integenic
H7	23	817224	-	XP_016273025.1	hypothetical protein
H10	18	786364	-	EGU11340.1	hypothetical protein
H11	17	73230	+	XP_016271631.1	monovalent inorganic cation transporter
H12	11	526329	+	XP_016274863.1	Set1C complex subunit Swd1

Note:

[a] Flanking sequence obtained from the corresponding number of T-DNA transformant

[b] T-DNA location was represented by the whole genome sequencing (*R. glutinis* ATCC 204091) scaffold number (**Scaffold**), position in the scaffold (**Position**) and direction of LB in the T-DNA (**direction**), where + and – represents the direction towards 3’ and 5’ of the scaffold sequence

[c] Best hit denotes the BLASTx result with the highest E-score

[d] Annotations were determined according to the BLASTx results

[e] Intergenic represents no valid gene affected
