# Supplementary material for: Identification of novel genes in the carotenogenic and oleaginous yeast Rhodotorula toruloides through genome-wide insertional mutagenesis
Source: BMC Microbiol. 2018 Feb 21;18:14. doi: 10.1186/s12866-018-1151-6 (PMC5822628; doi:10.1186/s12866-018-1151-6)
Supplement: Supplementary file 3 — Table S2. Summary of 192 T-DNA flanking sequences in the R. glutinis ATCC 204091 genome. (PDF 159 kb) [file 12866_2018_1151_MOESM3_ESM.pdf]

Additional file 3: Table S2. Summary of 192 T-DNA flanking sequences in the *R. glutinis* ATCC 204091 genome

Sequence code [a]	Genic site [b,c]	Best hit [d]	Annotation [e]	Organism [f]	Identity [g]
**LB sequences**					
A12	Genic	CGB_A8370W	MFS Transporter	*Cryptococcus bacillisporus*	72%
B12	Intergenic				
C12	Genic	XP_002395609.1	chitin synthase 6	*Moniliophthora perniciosa*	44%
A01	Genic	ZP_08358545.1	Beta-D-glucuronosideglucuronosohydrolase	*Escherichia coli*	96%
B01	Genic	ZP_08358545.1	Beta-D-glucuronosideglucuronosohydrolase	*Escherichia coli*	96%
D01	Genic	XP_001513359.2	FK506-binding protein 2B-like	*Ornithorhynchus anatinus*	39%
F01	Genic	YP_001433685.1	ArsR family transcriptional regulator	*Roseiflexus castenholzii*	30%
H01	Upstream 0.5 kb	XP_660959.1	amidase	*Aspergillus nidulans*	49%
A02	Genic	ZP_09452338.1	translation initiation factor IF-2	*Lactobacillus zeae*	38%
B02	Genic	YP_159354.1	pantothenate kinase	*Aromatoleum aromaticum*	37%
C02	Genic	XP_002640005.1	Hypothetical protein	*Caenorhabditis briggsae*	53%
D02	Upstream 0.5 kb	XP_002484838.1	conserved hypothetical protein	*Talaromyces stipitatus*	38%
E02	Upstream 1.0 kb	XP_001261866.1	C6 zinc finger domain protein	*Neosartorya fischeri*	57%
G02	Genic	XP_002367651.1	hypothetical protein	*Toxoplasma gondii*	33%
H02	Genic	ZP_07628725.1	putative lipoprotein	*Prevotella amnii*	45%
A03	Genic	XP_002413381.1	hypothetical protein	*Ixodes scapularis*	33%
E03	Genic	YP_005261613.1	polyketide synthase	*Nocardia cyriacigeorgica*	38%
F03	Genic	XP_003325975.1	NADH-ubiquinone oxidoreductase	*Puccinia graminis*	82%
G03	Genic	XP_002411805.1	hypothetical protein	*Ixodes scapularis*	39%

B04	Upstream 0.5 kb	GAA97286.1|	hypothetical protein	*Mixia osmundae*	36%
C04	Genic	EHK46547.1	hypothetical protein	*Trichoderma atroviride*	34%
D04	Upstream 0.5 kb	EFA12702.1	hypothetical protein	*Tribolium castaneum*	35%
E04	Genic	XP_001702651.1	flagellar associated protein	*Chlamydomonas reinhardtii*	36%
F04	Genic	EFX76696.1	hypothetical protein	*Daphnia pulex*	39%
G04	Genic	ZP_06970446.1	transposase	*Ktedonobacter racemifer*	40%
A05	Downstream 0.3 kb	XP_003334153.1	transcriptional activator protein copR	*Puccinia graminis*	41%
B05	Genic	CAA08846.1	DNA integrase	*Salmonella typhimurium*	100%
C05	Downstream 0.3 kb	EFP85564.2	hypothetical protein PGTG_11920	*Puccinia graminis*	29%
D05	Upstream 0.5 kb	EFZ00710.1	hypothetical protein MAA_03306	*Metarhizium anisopliae*	37%
E05	Upstream 0.5 kb	YP_006365479.1	hypothetical protein MODMU_1541	*Modestobacter marinus*	44%
F05	Upstream 0.5 kb	EFW79359.1	hypothetical protein PsgB076_17966	*Pseudomonas syringae*	31%
G05	Genic	EFP85564.2	hypothetical protein PGTG_11920	*Puccinia graminis*	29%
H05	Genic	EFP85564.2	hypothetical protein PGTG_11920	*Puccinia graminis*	29%
A06	Genic	ZP_09648351.1	DNA/RNA helicase, superfamily II	*Bradyrhizobium sp. WSM471*	40%
B06	Genic	XP_003071652.1	hygromycin-B	*Coccidioides posadasii*	45%
C06	Genic	XP_756991.1	succinate dehydrogenase	*Ustilago maydis*	98%
E06	Genic	NP_587782.1	argonaute	*Schizosaccharomyces pombe*	44%
G06	Upstream 0.5 kb	YP_900144.1	molybdenum cofactor synthesis domain-containing protein	*Pelobacter propionicus*	41%
H06	Upstream 0.5 kb	YP_004303810.1	Biotin-(acetyl-CoA-carboxylase) ligase	*Polymorphum gilvum*	33%
C07	Genic	XP_001936573.1	smad nuclear interacting protein 1	*Pyrenophora tritici-repentis*	44%
D07	Genic	XP_001692436.1	dicer-like protein	*Chlamydomonas reinhardtii*	35%

E07	Upstream 0.5 kb	XP_001497160.1	keratin-associated protein 9-7-like	*Equus caballus*	33%
G07	Upstream 0.5 kb	XP_003191693.1	sulfate transporter	*Cryptococcus gattii*	69%
H07	Genic	YP_706162.1	error-prone DNA polymerase	*Rhodococcus jostii*	33%
A08	Genic	ZP_07628725.1	putative lipoprotein	*Prevotella amnii*	45%
B08	Genic	XP_002395609.1	chitin synthase 6	*Moniliophthora perniciosa*	45%
C08	Genic	ZP_08358545.1	beta-glucuronidase (GUS) Beta-D-glucuronosideglucuronosohydrolase	*Escherichia coli*	76%
D08	Upstream 0.5 kb	XP_003037290.1	hypothetical protein SCHCODRAFT_64476	*Schizophyllum commune*	52%
E08	Genic	XP_001513359.2	FK506-binding protein 2B-like	*Ornithorhynchus anatinus*	41%
F08	Genic	XP_001936845.1	conserved hypothetical protein	*[Pyrenophora tritici-repentis Pt-1C-BFP*	44%
G08	Upstream 0.5 kb	XP_003035588.1	Amidase	*Schizophyllum commune*	42%
H08	Genic	ZP_09452338.1	translation initiation factor IF-2	*Lactobacillus zeae*	38%
A09	Downstream 0.3 kb	YP_159354.1	pantothenate kinase	*Aromatoleum aromaticum*	36%
B09	Genic	XP_002640005.1	Hypothetical protein CBG12474	*Caenorhabditis briggsae*	53%
G09	Upstream 0.5 kb	ZP_06733063.1	Site-specific recombinase	*Xanthomonas fuscans*	34%
H09	Upstream 0.5 kb	ZP_09803704.1	putative hydrolase	*Mobilicoccus pelagius*	31%
A10	Upstream 0.5 kb	XP_003325975.1	NADH-ubiquinone oxidoreductase	*Puccinia graminis*	83%
B10	Genic	XP_002411805.1	hypothetical protein IscW_ISCW021794	*Ixodes scapularis*	41%
D10	Genic	XP_003169193.1	hypothetical protein MGYG_08739	*Arthroderma gypseum*	40%
F10	Genic	YP_004285231.1	hypothetical protein ACMV_30020	*Acidiphilium multivorum*	35%
G10	Genic	YP_005467423.1	hypothetical protein	*Actinoplanes missouriensis*	53%
H10	Upstream 1.0 kb	ZP_10083140.1	hypothetical protein Bra1253DRAFT_03911	*Bradyrhizobium sp.*	36%

A11	Genic	ZP_09648351.1	DNA/RNA helicase, superfamily II	*Bradyrhizobium sp.*	40%
B11	Genic	XP_756991.1	succinate dehydrogenase	*Ustilago maydis*	85%
C11	Genic	NP_587782.1	argonaute	*Schizosaccharomyces pombe*	44%
E11	Upstream 0.5 kb	XP_001839083.2	peptidyl-prolyl cis-trans isomerase-like 4	*Coprinopsis cinerea*	64%
F11	Genic	XP_003007824.1	predicted protein	*Verticillium albo-atrum*	29%
G11	Genic	XP_001692436.1	dicer-like protein	*Chlamydomonas reinhardtii*	35%
H11	Genic	XP_003055314.1	predicted protein	*Micromonas pusilla*	48%
A12	Upstream 0.5 kb	XP_003191693.1	transporter	*Cryptococcus gattii*	72%
B12	Genic	NP_001146516.1	uncharacterized protein LOC100280106	*Zea mays*	61%
C12	Genic	XP_002395609.1	hypothetical protein MPER_04314	*Moniliophthora perniciosa*	44%

RB sequences

P1A1	Upstream 0.5 kb	EGU11748.1	Nonribosomal peptide synthetase 4	*R. glutinis ATCC 204091*	100%
P1A1	Upstream 0.5 kb	EGU11748.1	Nonribosomal peptide synthetase 4	*R. glutinis ATCC 204091*	100%
P1A2	Genic	EGU12718.1	GMC oxidoreductase	*R. glutinis ATCC 204091*	88%
P1A3	Genic	EGU11448.1	Hypothetical protein RTG_02607	*R. glutinis ATCC 204091*	38%
P1A4	Genic	EGU12184.1	Proteophosphoglycan 5	*R. glutinis ATCC 204091*	100%
P1A5	Intergenic	EIM21194.1	Ras-related GTP-binding protein-like protein raga	*Wallemia sebi*	31%
P1A6	Genic	EGU12296.1	Proteophosphoglycan 4	*R. glutinis ATCC 204091*	31%
P1A7	Genic	EGU13389.1	Reverse transcriptase-RNase H-integrase	*R. glutinis ATCC 204091*	32%
P1A8	Upstream 1.0 kb	EGU11055.1	CAMK/CAMKL/PASK protein kinase	*R. glutinis ATCC 204091*	100%
P1A9	Downstream 0.3 kb	EGF97645.1	hypothetical protein MELLADRAFT_79816	*Melampsora larici-populina*	35%

P1A10	Upstream 0.5 kb	XP_001385993.1	DNA-directed RNA polymerase II	*Scheffersomyces stipitis*	58%
P1B1	Upstream 0.5 kb	EGU11748.1	Nonribosomal peptide synthetase 4	*R. glutinis ATCC 204091*	100%
P1B2	Intergenic	EGU12701.1	Ubiquitin carrier protein	*R. glutinis ATCC 204091*	34%
P1B3	Genic	EGU12184.1	Proteophosphoglycan 5	*R. glutinis ATCC 204091*	100%
P1B6	Upstream 0.5 kb	EGU11827.1	Proteophosphoglycan ppg4	*R. glutinis ATCC 204091*	58%
P1B7	Genic	XP_003197329.1	Formaldehyde dehydrogenase (glutathione-dependent)	*Cryptococcus gattii*	43%
P1B8	Downstream 0.3 kb	EGU12466.1	ATP dependent helicase	*R. glutinis ATCC 204091*	33%
P1C1	Unknown				
P1C2	Unknown				
P1C3	Genic	EGU12184.1	Proteophosphoglycan 5	*R. glutinis ATCC 204091*	100%
P1C4	Genic	EGU13548.1	Succinate-semialdehyde dehydrogenase	*R. glutinis ATCC 204091*	99%
P1C5	Genic	EGU12184.1	Proteophosphoglycan 5	*R. glutinis ATCC 204091*	100%
P1C9	Intergenic	XP_566492.1	Vacuole protein	*Cryptococcus neoformans*	44%
P1C10	Upstream 0.5 kb	EGU11400.1	Endo-polygalacturonase PG1	*R. glutinis ATCC 204091*	78%
P1C11	Intergenic	AFR93439.1	5-aminolevulinate synthase	*Cryptococcus neoformans*	63%
P1D2	Intergenic	EGU12701.1	Ubiquitin carrier protein	*R. glutinis ATCC 204091*	34%
P1D3	Genic	EGU11312.1	Sensitive to high expression protein 9	*R. glutinis ATCC 204091*	99%
P1D4	Genic	EGU13548.1	Succinate-semialdehyde dehydrogenase	*R. glutinis ATCC 204091*	99%
P1D6	Genic	EGU12184.1	Proteophosphoglycan 5	*R. glutinis ATCC 204091*	100%
P1D7	Intergenic	EIM21194.1	Ras-related GTP-binding protein-like protein raga	*Wallemia sebi CBS 633.66*	31%
P1D8	Unknown				
P1D9	Upstream 1.0 kb	AFR95571.1	GTP-binding nuclear protein GSP1/Ran	*Cryptococcus neoformans*	70%
P1D10	Upstream 0.5 kb	EGU13101.1	Amidohydrolase family protein	*R. glutinis ATCC 204091*	99%

P1D12	Genic	EGU10835.1	Proteophosphoglycan ppg4	*R. glutinis ATCC 204091*	72%
P1E1	Upstream 1.0 kb	EGU13177.1	Septin	*R. glutinis ATCC 204091*	58%
P1E3	Genic	EGU11477.1	Vacuolar fusion protein MON1	*R. glutinis ATCC 204091*	99%
P1E4	Genic	EGU13548.1	Succinate-semialdehyde dehydrogenase	*R. glutinis ATCC 204091*	99%
P1E6	Genic	EGU10896.1	UTP-glucose-1-phosphate uridylyltransferase	*R. glutinis ATCC 204091*	100%
P1E8	Genic	EGU10835.1	Proteophosphoglycan ppg4	*R. glutinis ATCC 204091*	72%
P1E11	Downstream 0.3 kb	EGU13636.1	Acetate kinase	*R. glutinis ATCC 204091*	100%
P1E12	Genic	XP_569369.1	Fatty acid elongase	*Cryptococcus neoformans*	44%
P1F1	Genic	EGU12184.1	Proteophosphoglycan 5	*R. glutinis ATCC 204091*	100%
P1F2	Upstream 0.5 kb	EGU11486.1	Hypothetical protein RTG_02656	*R. glutinis ATCC 204091*	29%
P1F4	Genic	EGU11134.1	Delta 8-sphingoloid desaturase protein	*R. glutinis ATCC 204091*	95%
P1F5	Genic	EGU11134.1	Delta 8-sphingoloid desaturase protein	*R. glutinis ATCC 204091*	95%
P1F6	Genic	EGU11846.1	Proteophosphoglycan ppg4	*R. glutinis ATCC 204091*	100%
P1F8	Genic	EGU12184.1	Proteophosphoglycan 5	*R. glutinis ATCC 204091*	100%
P1F9	Upstream 0.5 kb	EGU11109.1	Beta-glucosidase	*R. glutinis ATCC 204091*	79%
P1F10	Upstream 0.5 kb	XP_806376.1	Hypothetical protein	*Trypanosoma cruzi*	41%
P1F11	Upstream 0.5 kb	XP_806376.1	Hypothetical protein	*Trypanosoma cruzi*	41%
P1F12	Genic	EGU13282.1	Hypothetical protein RTG_00445	*R. glutinis ATCC 204091*	75%
P1G2	Genic	EGU11865.1	Proteophosphoglycan 5	*R. glutinis ATCC 204091*	92%
P1G3	Genic	EGU11875.1	Hypothetical protein RTG_02120	*R. glutinis ATCC 204091*	100%
P1G5	Genic	EGU10896.1	UTP-glucose-1-phosphate uridylyltransferase	*R. glutinis ATCC 204091*	100%
P1G6	Genic	EGU11389.1	Hypothetical protein RTG_02544	*R. glutinis ATCC 204091*	66%
P1G8	Genic	EGF99466.1	Zinc metalloprotease	*Melampsora larici-populina*	43%

P1G9	Genic	EGU13267.1	Chromatin modification-related protein EAF3	*R. glutinis ATCC 204091*	94%
P1G10	Genic	EJD43690.1	Cation efflux protein	*Auricularia delicata*	54%
P1G12	Genic	EGU13282.1	Hypothetical protein RTG_00445	*R. glutinis ATCC 204091*	75%
P1H1	Genic	EGU12184.1	Proteophosphoglycan 5	*R. glutinis ATCC 204091*	100%
P1H2	Genic	EGU11865.1	Proteophosphoglycan 5	*R. glutinis ATCC 204091*	92%
P1H3	Genic	EIN03812.1	P-loop containing nucleoside triphosphate hydrolase protein	*Punctularia strigosozonata*	74%
P1H4	Genic	EGU12184.1	Proteophosphoglycan 5	*R. glutinis ATCC 204091*	100%
P1H5	Downstream 0.3 kb	XP_569415.1	60S ribosomal protein	*Cryptococcus neoformans*	70%
P1H7	Genic	EGU13568.1	Alpha-detoglutarate dependent xanthine dioxygenase	*R. glutinis ATCC 204091*	95%
P1H8	Genic	EGU11250.1	STE/STE20/FRAY protein kinase	*R. glutinis ATCC 204091*	95%
P1H12	Genic	EGU12528.1	RNA polymerase II transcription factor	*R. glutinis ATCC 204091*	63%
P2A2	Genic	EGU12695.1	Transcription factor IF-2	*R. glutinis ATCC 204091*	33%
P2A4	Genic	EGU11658.1	Proteophosphoglycan 5	*R. glutinis ATCC 204091*	84%
P2A6	Genic	EGU12184.1	Proteophosphoglycan 5	*R. glutinis ATCC 204091*	100%
P2A7	Genic	EGU12184.1	Proteophosphoglycan 5	*R. glutinis ATCC 204091*	100%
P2A10	Genic	EGU12184.1	Proteophosphoglycan 5	*R. glutinis ATCC 204091*	100%
P2A11	Genic	EGU12184.1	Proteophosphoglycan 5	*R. glutinis ATCC 204091*	100%
P2A12	Genic	XP_002911253.1	Hypothetical protein CC1G_14682	*Coprinopsis cinerea*	30%
P2B1	Genic	EGU11681.1	Ribosomal RNA-processing protein 8	*R. glutinis ATCC 204091*	99%
P2B3	Intergenic	EJU04364.1	Hypothetical protein DACRYDRAFT_14463	*Dacryopinax sp. DJM-731 SS1*	47%
P2B5	Genic	EGU11448.1	Hypothetical protein RTG_02607	*R. glutinis ATCC 204091*	38%
P2B6	Genic	EGG03372.1	Hypothetical protein MELLADRAFT_109351	*Melampsora larici-populina*	27%
P2B7	Genic	EGU11192.1	Transcription factor Hsf1	*R. glutinis ATCC 204091*	36%

P2B8	Intergenic	CBQ69926.1	Conserved hypothetical protein	*Sporisorium reilianum SRZ2*	35%
P2B9	Intergenic	CBQ69926.1	Conserved hypothetical protein	*Sporisorium reilianum SRZ2*	35%
P2B10	Unknown				
P2B12	Upstream 1.0 kb	EGU11340.1	Hypothetical protein RTG_02812	*R. glutinis ATCC 204091*	98%
P2C1	Upstream 1.0 kb	EGU12878.1	Putative DNA helicase INO80	*R. glutinis ATCC 204091*	99%
P2C2	Upstream 1.0 kb	EGU12261.1	Hypothetical Protein RTG_01639	*R. glutinis ATCC 204091*	75%
P2C3	Genic	XP_756979.1	Hypothetical protein UM00832.1	*Ustilago maydis*	31%
P2C4	Upstream 0.5 kb	EGU12879.1	Proteophosphoglycan 5	*R. glutinis ATCC 204091*	99%
P2C7	Genic	EGU13281.1	Hypothetical protein RTG_00444	*R. glutinis ATCC 204091*	99%
P2C10	Genic	EGU12184.1	Proteophosphoglycan 5	*R. glutinis ATCC 204091*	100%
P2C11	Genic	EGU12184.1	Proteophosphoglycan 5	*R. glutinis ATCC 204091*	100%
P2C12	Upstream 0.5 kb	XP_806376.1	Hypothetical protein	*Trypanosoma cruzi*	41%
P2D1	Genic	EGU10835.1	Proteophosphoglycan ppg4	*R. glutinis ATCC 204091*	72%
P2D5	Upstream 0.5 kb	EIW61980.1	DnaJ-domain-containing protein	*Trametes versicolor*	44%
P2D6	Upstream 0.5 kb	EMD32447.1	Hypothetical protein CERSUDRAFT_126841	*Ceriporiopsis subvermispora*	58%
P2D7	Intergenic	EGU11306.1	DNA repair protein rad5	*R. glutinis ATCC 204091*	82%
P2D8	Upstream 1.0 kb	XP_003197134.1	EF-hand calcium-binding protein, Caltractin-cdc31 subfamily	*Cryptococcus gattii*	53%
P2D9	Upstream 1.0 kb	XP_003197134.1	EF-hand calcium-binding protein, Caltractin-cdc31 subfamily	*Cryptococcus gattii*	53%
P2D10	Genic	EGU12184.1	Proteophosphoglycan 5	*R. glutinis ATCC 204091*	100%
P2D11	Unknown				
P2E4	Genic	EIM80360.1	NCS1 nucleoside transporter	*Stereum hirsutum*	60%
P2E5	Upstream 1.0 kb	XP_003321176.2	Actin-like protein 2	*Puccinia graminis*	70%
P2E6	Genic	EGU11250.1	STE/SET20/FRAY protein kinase	*R. glutinis ATCC 204091*	95%

P2E10	Upstream 1.0 kb	XP_003890326.1	ATP citrate lyase	*Puccinia graminis*	60%
P2E11	Genic	EGU12184.1	Proteophosphoglycan 5	*R. glutinis ATCC 204091*	100%
P2E12	Genic	EGU12184.1	Proteophosphoglycan 5	*R. glutinis ATCC 204091*	100%
P2F1	Unknown				
P2F2	Upstream 0.5 kb	EGU12138.1	Hypothetical Protein RTG_01751	*R. glutinis ATCC 204091*	72%
P2F3	Genic	EGU12148.1	Putative transporter	*R. glutinis ATCC 204091*	48%
P2F4	Upstream 0.5 kb	EGU11504.1	GTP-binding protein RAB5	*R. glutinis ATCC 204091*	100%
P2F5	Genic	EJD41783.1	Opi1-domain-containing protein	*Auricularia delicata*	36%
P2F8	Unknown				
P2F10	Upstream 1.0 kb	EGU11060.1	Biotin/lipoyl attachment:Carbamoyl-phosphate synthase subunit L	*R. glutinis ATCC 204091*	48%
P2G2	Genic	EGU10835.1	Proteophosphoglycan ppg4	*R. glutinis ATCC 204091*	72%
P2G3	Genic	EJC98857.1	Pyruvate decarboxylase	*Fomitiporia mediterranea*	38%
P2G4	Genic	EGU11102.1	ABC transporter	*R. glutinis ATCC 204091*	100%
P2G5	Genic	EGU11264.1	Mitochondrial import inner membrane translocase subunit tim23	*R. glutinis ATCC 204091*	46%
P2G6	Unknown				
P2G7	Genic	EGU12987.1	Proteophosphoglycan ppg4	*R. glutinis ATCC 204091*	41%
P2G8	Genic	EGU13623.1	Phosphotransferase family protein	*R. glutinis ATCC 204091*	99%
P2G9	Upstream 0.5 kb	EGU13623.1	Phosphotransferase family protein	*R. glutinis ATCC 204091*	99%
P2G11	Genic	EIW73372.1	Transcription factor RfeF TREMEDRAFT_56189	*Tremella mesenterica*	50%
P2G12	Genic	EGU13198.1	Hypothetical protein RTG_00359	*R. glutinis ATCC 204091*	93%
P2H1	Downstream 0.3 kb	EGU13576.1	Serine/threonine protein kinase	*R. glutinis ATCC 204091*	100%
P2H3	Unknown				
P2H4	Unknown				

P2H5	Genic	EEE61011.1	Hypothetical protein OsJ_14831	*Oryza sativa Japonica*	33%
P2H6	Upstream 0.5 kb	XP_003197147.1	NADH-ubiquinone oxidoreductase	*Cryptococcus gattii*	70%
P2H7	Upstream 1.0 kb	EGU13453.1	Helicase SWR1	*R. glutinis ATCC 204091*	100%
P2H8	Genic	EGU11734.1	Putative metallo-beta-lactamase	*R. glutinis ATCC 204091*	91%
P2H9	Genic	EGU11734.1	Putative metallo-beta-lactamase	*R. glutinis ATCC 204091*	91%
P2H11	Genic	EGU12184.1	Proteophosphoglycan 5	*R. glutinis ATCC 204091*	100%
P2H12	Upstream 1.0 kb	XP_003201506.1	Calcium-dependent secretion activator 1	*Danio rerio*	29%

[a] Flanking sequence obtained from corresponding to number of T-DNA transformant

[b] T-DNA tagged genes were determined according to the BLASTx results

[c] Upstream 1.0 kb, Upstream 0.5 kb and downstream 0.3 kb denotes T-DNA insertions within upstream 501~1000 bp, 500 bp and downstream 300 bp of the corresponding tagged gene, respectively

[d] Best hit denotes the BLASTx result with the highest E-score

[e] Annotations were determined according to the BLASTx results

[f] Microorganism denotes the host of Best hit

[g] Identity value was from BLASTx result
