# Supplementary material for: Identification of novel genes in the carotenogenic and oleaginous yeast Rhodotorula toruloides through genome-wide insertional mutagenesis
Source: BMC Microbiol. 2018 Feb 21;18:14. doi: 10.1186/s12866-018-1151-6 (PMC5822628; doi:10.1186/s12866-018-1151-6)
Supplement: Supplementary file 4 — Figure S2. Chemical-assisted and visual screening systems. (A) Cerulenin-assisted screening for high PUFA producing mutants. - and + represents the absence and presence of 50 μg/mL cerulenin, respectively. (B) Nile red-assisted screening for high lipid producing mutants. L and H represents the low and high fluorescence intensity on 0.5 μg/mL nile red-containing YPD agar, respectively. (C) Tetrazeolium violet-assisted screening for high lipid producing mutants. L and H represents the low and high violet intensity on 10 μg/mL tetrazolium violet-containing YPD agar, respectively. (D) Visual screening for carotenoid producing mutants (indicated by arrow heads). (PDF 854 kb) [file 12866_2018_1151_MOESM4_ESM.pdf]

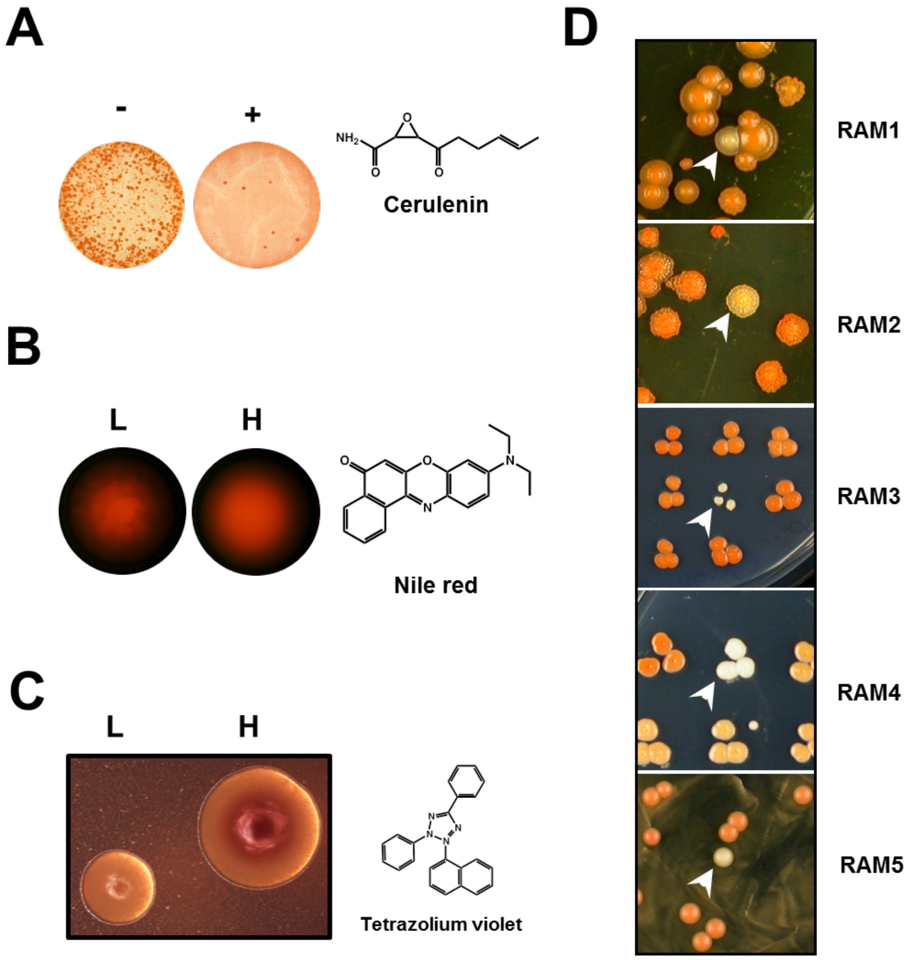

Additonal file 4: Fig. S2. Chemical-assisted and visual screening systems. (A) Cerulenin-assisted screening for high PUFA producing mutants. - and + represents the absence and presence of 50 µg/mL cerulenin, respectively. (B) Nile red-assisted screening for high lipid producing mutants. L and H represents the low and high fluorescence intensity on 0.5 µg/mL nile red-containing YPD agar, respectively. (C) Tetrazeolium violet-assisted screening for high lipid producing mutants. L and H represents the low and high violet intensity on 10 µg/mL tetrazolium violet-containing YPD agar, respectively. (D) Visual screening for carotenoid producing mutants (indicated by arrow heads).
