# Supplementary material for: Identification of novel genes in the carotenogenic and oleaginous yeast Rhodotorula toruloides through genome-wide insertional mutagenesis
Source: BMC Microbiol. 2018 Feb 21;18:14. doi: 10.1186/s12866-018-1151-6 (PMC5822628; doi:10.1186/s12866-018-1151-6)
Supplement: Supplementary file 5 — Table S3. Sequences of oligonucleotides. (PDF 75 kb) [file 12866_2018_1151_MOESM5_ESM.pdf]

Additional file 5: Table S3. Sequences of oligonucleotides

Name	Sequence (5’-3’)	Restriction Site	Purpose
**<u>Promoter amplification</u>**			
Pgap-Sf	TTT*actagt*AGATCTTGCTGATAGGCAGGT	SpeI	U. maydis *gpd1* promoter
Pgap-Nr	TTT*ccatgg*GAAGAGTGTTTTGGTTTCGAA	NcoI	
PgpdA-Sf	TTT*actagt*CTGTACAGTGACCGGTGAC	SpeI	*A. nidulans gpdA* promoter
PgpdA-Nr	TCA*ccatgg*TGATGTCTGCTCAAG	NcoI	
Ptef-Sf	TTT*actagt*CAGCGACATGGAGGCCCAG	SpeI	*A. gossypii tefA* promoter
Ptef-Nr	TAC*ccatgg*TTGTTTATGTTCGGATG	NcoI	
Rt011S	TTT*actagt*CTGCAGAACTACGCCCTCTC	SpeI	Rt *GPD1* promoter
Rt012N	TTT*ccatgg*TGAGTGATCTGGTGTTGTTC	NcoI	
**<u>Hi-TAIL PCR</u>**			
HRSP1	GAATCCTGTTGCCGGTCTTGCGATG		
HRSP2	TTATGATTAGAGTCCCGCAATTATACA		
HRSP3	CTAGCTTAGCTTGAGCTTGGATC		
HRRSP1	GTGCTGACGCGGGCATAGCCCAG		
HRRSP2	ATGCGACTAAAACACGCGACAAGA		
HRRSP3	AGCAGCGGAGGGGTTGGATC		
LAD1-1	ACGATGGACTCCAGAGCGGCCGC(GCA)N(GCA)NNNGGAA		
LAD1-4	ACGATGGACTCCAGAGCGGCCGC(GCT)(GAT)N(GCT)NNNCGGT		
M13FP	GTAAAACGACGGCCAGT		
M13RP	CAGGAAACAGCTATGAC		
**<u>Deletion, qPCR and complementation of *CAR1*</u>**			
Rt128-2	CAGGCCTTCGCCATCGGATT		Gene

Rt127-2	GGAACTCATCCGCTCGATCG	targeted deletion – *CAR1*
Rt141	CTTTCCGACCGACTTCTTGCT	DIG-probe – *CAR1*
Rt140	CGCTGACCTTCCCAATCTTTC	
Rt319Sf	TTTACTAGTCGGAACGAGGCGCAATG	*CAR1* allele
Rt128-2	CAGGCCTTCGCCATCGGATT	
qCAR1f	GCAAGATACCCCAGCTCGAC	qPCR of *CAR1*
qCAR1r	GGGGACGTTGACGTAGAAGG	
qACT1f	TACCCAACTTGTCCCAACCTG	qPCR of *ACT1*
qACT1r	CTCGTCTCCATCACCATCCTC	
