# Supplementary material for: Identification of novel genes in the carotenogenic and oleaginous yeast Rhodotorula toruloides through genome-wide insertional mutagenesis
Source: BMC Microbiol. 2018 Feb 21;18:14. doi: 10.1186/s12866-018-1151-6 (PMC5822628; doi:10.1186/s12866-018-1151-6)
Supplement: Supplementary file 6 — Figure S3. T-DNA organizations in contructs used in this study. All binary vectors have the same pPZP200 backbone [84]. (A) pEC3Pxxx-HPT3. (B) pEC3GPD-GUS. LB: left border of T-DNA; RB: right border of T-DNA; Pxxx represents three glyceraldehydes-3-phosphate dehydrogenase promoters from A. nidulans (PgpdA), U. maydis (Pgpd) and R. toruloides (PGPD1) and the tranlation elongation factor promoter from A. gossypii (Ptef). hpt-3: codon-optimized hygromycin resistance gene based on the codon usage bias in R. toruloides; GUS: E. coli β-glucuronidase gene; T35S: terminator of cauliflower mosaic virus 35S gene; Tnos: terminator of A. tumefaciens nopaline synthase gene; Ttef: terminator of A. gossypii translation elongation factor gene; Tcyc1: Terminator of S. cerevisiae iso-1-cytochrome C gene. The labeled restriction enzymes are unique cutting sites in the plasmid. (PDF 56 kb) [file 12866_2018_1151_MOESM6_ESM.pdf]

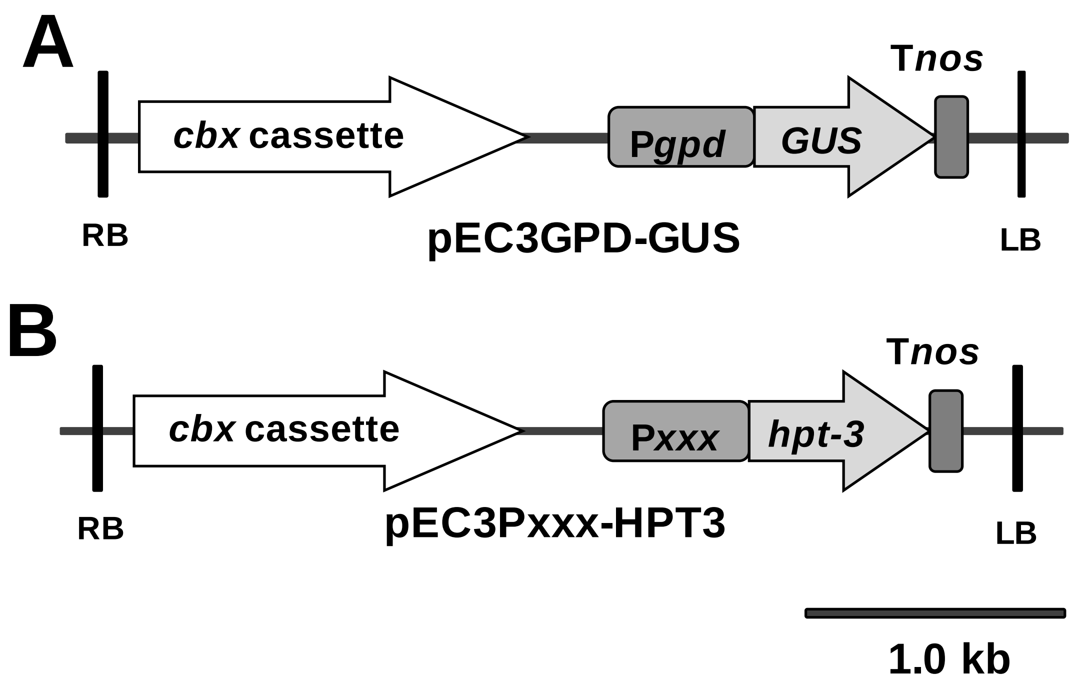

Additional file 6: Figure S3. T-DNA organizations in contructs used in this study. All binary vectors have the same pPZP200 backbone [84]. (A) pEC3Pxxx-HPT3. (B) pEC3GPD-GUS. LB: left border of T-DNA; RB: right border of T-DNA; P*xxx* represents three glyceraldehydes-3-phosphate dehydrogenase promoters from *A. nidulans* (P_{gpdA}), *U. maydis* (P_{gpd}) and *R. toruloides* (P_{GPD1}) and the tranlation elongation factor promoter from *A. gossypii* (P_{tef}). *hpt-3*: codon-optimized hygromycin resistance gene based on the codon usage bias in *R. toruloides*; GUS: *E. coli* β-glucuronidase gene; T_{35S}: terminator of cauliflower mosaic virus 35S gene; Tnos: terminator of *A. tumefaciens* nopaline synthase gene; T_{tef}: terminator of *A. gossypii* translation elongation factor gene; T_{cyc1}: Terminator of *S. cerevisiae* iso-1-cytochrome C gene. The labeled restriction enzymes are unique cutting sites in the plasmid.
